# Severe COVID-19 in Vaccinated Adults With Hematologic Cancers in the Veterans Health Administration

**DOI:** 10.1001/jamanetworkopen.2024.0288

**Published:** 2024-02-23

**Authors:** Sonia T. Anand, Austin D. Vo, Jennifer La, Nhan V. Do, Nathanael R. Fillmore, Mary Brophy, Westyn Branch-Elliman, Paul A. Monach

**Affiliations:** 1 VA Boston Cooperative Studies Program, Boston, Massachusetts; 2 Boston University School of Medicine, Boston, Massachusetts; 3 Department of Medicine, VA Boston Healthcare System, Boston, Massachusetts; 4 Harvard Medical School, Boston, Massachusetts; 5 Dana-Farber Cancer Institute, Boston, Massachusetts; 6 VA Boston Center for Healthcare Organization and Implementation Research, Boston, Massachusetts

## Abstract

**Importance:**

With SARS-CoV-2 transforming into an endemic disease and with antiviral treatments available, it is important to establish which patients remain at risk of severe COVID-19 despite vaccination.

**Objective:**

To quantify the associations of clinical and demographic variables with odds of severe COVID-19 among patients with hematologic cancers.

**Design, Setting, and Participants:**

This case-control study included all patients with hematologic malignant neoplasms in the national Veterans Health Administration (VHA) who had documented SARS-CoV-2 infection after vaccination. Groups of patients with severe (cases) vs nonsevere (controls) COVID-19 were compared. Data were collected between January 1, 2020, and April 5, 2023, with data on infection collected between January 1, 2021, and September 30, 2022. All patients with diagnostic codes for hematologic malignant neoplasms who had documented vaccination followed by documented SARS-CoV-2 infection and for whom disease severity could be assessed were included. Data were analyzed from July 28 to December 30, 2023.

**Exposures:**

Clinical (comorbidities, predominant viral variant, treatment for malignant neoplasm, booster vaccination, and antiviral treatment) and demographic (age and sex) variables shown in prior studies to be associated with higher or lower rates of severe COVID-19. Comorbidities included Alzheimer disease or dementia, chronic kidney disease, chronic obstructive pulmonary disease, diabetes, heart failure, and peripheral vascular disease.

**Main Outcome and Measures:**

The main outcome was severe COVID-19 compared with nonsevere SARS-CoV-2 infection. Severe COVID-19 was defined as death within 28 days, mechanical ventilation, or hospitalization with use of dexamethasone or evidence of hypoxemia or use of supplemental oxygen. Multivariable logistic regression was used to estimate the associations of demographic and clinical variables with the odds of severe COVID-19, expressed as adjusted odds ratios (aORs) with 95% CIs.

**Results:**

Among 6122 patients (5844 [95.5%] male, mean [SD] age, 70.89 [11.57] years), 1301 (21.3%) had severe COVID-19. Age (aOR per 1-year increase, 1.05; 95% CI, 1.04-1.06), treatment with antineoplastic or immune-suppressive drugs (eg, in combination with glucocorticoids: aOR, 2.32; 95% CI, 1.93-2.80), and comorbidities (aOR per comorbidity, 1.35; 95% CI, 1.29-1.43) were associated with higher odds of severe disease, whereas booster vaccination was associated with lower odds (aOR, 0.73; 95% CI, 0.62-0.86). After oral antiviral drugs became widely used in March 2022, 20 of 538 patients (3.7%) with SARS-CoV-2 infection during this period had progression to severe COVID-19.

**Conclusions and Relevance:**

In this case-control study of patients with hematologic cancers, odds of severe COVID-19 remained high through mid-2022 despite vaccination, especially in patients requiring treatment.

## Key Points

**Question:**

What are the odds of COVID-19 becoming severe in patients with hematologic malignant neoplasms who have been vaccinated?

**Findings:**

In this case-control study including 6122 vaccinated patients with hematologic cancers and SARS-CoV-2 infection, 1301 patients (21.3%) developed severe COVID-19. Age, comorbidities, and treatment for malignant neoplasm were associated with higher odds of severe COVID-19.

**Meaning:**

These findings suggest that prevalence of severe COVID-19 among patients with SARS-CoV-2 infection remains high in those with hematologic cancers despite vaccination and is associated with a combination of cancer-related and other variables.

## Teaser

This case-control study assesses the associations of clinical and demographic variables with odds of severe COVID-19 among patients with hematologic cancers in the Veterans Health Administration.

## Introduction

Patients with hematologic cancers are at higher risk for severe COVID-19 and death than the general population, even when following standard mRNA vaccination regimens, including booster or additional vaccine doses.^1,2,3^ Studies have demonstrated ongoing elevated risk of severe COVID-19 among patients coded as having hematologic malignant neoplasms, despite vaccination and boosting.^2,4,5,6,7,8,9,10,11,12,13^ In our previous study of more than 110 000 US veterans with SARS-CoV-2 infection after vaccination, the broad set of *International Statistical Classification of Diseases and Related Health Problems, Tenth Revision* (*ICD-10*) codes for leukemias or lymphomas was associated with increased frequency of severe COVID-19 (adjusted odds ratio [aOR], 1.58; 95% CI, 1.33-1.88). Cytotoxic chemotherapy and different classes of immune-suppressive drugs, including 2 that are often used in treatment of hematologic malignant neoplasms (glucocorticoids and lymphocyte-depleting drugs), also were associated with increased frequency of severe disease. A meta-analysis of studies focused on hematologic malignant neoplasms reported larger effect sizes (relative risk for hospitalization, 2.54; relative risk for death, 2.88), but the comparison was vs controls without malignant neoplasms.^6^

Our previous study and most others have lumped different hematologic malignant neoplasms together^5,7^ or have focused on 1 disease. The aim of this study is to quantify risk of severe COVID-19 among US veterans with SARS-CoV-2 infection following vaccination and with 1 of 8 different classes of hematologic malignant and premalignant conditions and to analyze clinical and demographic variables associated with severe disease.

## Methods

This case-control study was approved by the VA Boston Healthcare System Research and Development Committee as an exempt study prior to data collection and analysis, with a waiver of informed consent per 38 CFR 16.104d(4)iii. This report follows the Strengthening the Reporting of Observational Studies in Epidemiology (STROBE) reporting guideline.

### Cohort Creation

Inclusion criteria were diagnosis of a hematologic cancer, documented completion of an initial SARS-CoV-2 vaccine series, documented SARS-CoV-2 infection after vaccination, and sufficient data to assess severity of that infection. No comparison was made with patients without hematologic cancer, patients without vaccination, or patients without infection. First, all patients in the US Veterans Health Administration (VHA) with documented completion of an initial SARS-CoV-2 vaccine series (2 doses of an mRNA vaccine or 1 dose of an adenovirus vaccine) prior to December 1, 2021, and with subsequent documentation of SARS-CoV-2 infection by September 30, 2022, were identified. The cohort was then limited to patients coded as having hematologic malignant neoplasms. Malignant neoplasms were classified as lymphoid and myeloid, with lymphoid further subdivided into Hodgkin lymphoma (HL), non-Hodgkin lymphoma (NHL), chronic lymphocytic leukemia (CLL), acute lymphocytic leukemia, and plasmacytoid (PC) malignant neoplasms. Myeloid malignant neoplasms were subdivided into myeloproliferative disorders (MPD), myelodysplastic syndromes (MDS), chronic myelogenous leukemia (CML), and acute myeloblastic leukemia (AML). Analysis was not performed if a subgroup had fewer than 20 patients. We did not attempt more granular subtyping, such as separating the diverse subtypes of NHL or distinguishing Waldenstrom macroglobulinemia from multiple myeloma. Patients with diagnoses in both myeloid and lymphoid lineages were excluded as being likely to include coding errors. Patients with coding for multiple diagnoses solely within the myeloid or lymphoid subgroups were then classified based on the most advanced malignant neoplasm (in the following order: AML, CML, MDS, then MPD or PC, CLL, NHL, then HL). Patient demographic characteristics were extracted from VHA data, including self-reported race, categorized as American Indian or Alaska Native, Asian, Black or African American, Native Hawaiian or Other Pacific Islander, White, and unknown and self-reported ethnicity, categorized as Hispanic or Latino, not Hispanic or Latino, and unknown. Race and ethnicity data were included to describe the cohort but were not included in outcome analyses.

### Data Sources, Outcomes, and Definitions

Data were obtained from the VA COVID-19 Shared Data Resource and Corporate Data Warehouse encompassing the time period between January 1, 2020, and April 5, 2023. A complete list of definitions is included in eTable 1 in Supplement 1. Data for assessment of vaccination and SARS-CoV-2 infection were collected between December 15, 2020, and September 30, 2022.

As in our previous study,^4^ cases were patients with severe breakthrough SARS-CoV-2 infection, defined as requiring acute-care hospitalization within 14 days of the first documented positive test result for SARS-CoV-2 infection (polymerase chain reaction or antigen test) with documented blood oxygen level of less than 94% or receipt of supplemental oxygen (at any flow rate), use of dexamethasone or mechanical ventilation, or death of any cause 2 to 28 days after the positive test result. Controls were patients who met inclusion criteria and had nonsevere breakthrough SARS-CoV-2 infection, defined as either no hospitalization or hospitalization that did not meet criteria for severe disease.

Hematologic malignant neoplasms were defined by use of *ICD-10* codes from January 1, 2020, through April 5, 2023 (eTable 1 in Supplement 1). The time period for surveillance for these diagnoses was extended beyond infection to capture patients with delays between symptom onset and diagnosis, diagnosis at a non-VHA facility and code use at the VHA, or progression to a more malignant state. Recent treatment with antineoplastic or immune-suppressive medications prior to infection was classified as treatment with cytotoxic chemotherapy within 6 months preceding infection, lymphocyte-depleting therapy within 12 months, and/or treatment with other nonglucocorticoid antineoplastic or immune-suppressive drugs within 3 months; treatment with systemic glucocorticoids (prednisone or methylprednisolone) alone in the 3 months before infection; treatment with both glucocorticoids and other antineoplastic or immune-suppressive drugs; or none of these. Isolated systemic glucocorticoid use was kept separate because it could be used for some hematologic malignant neoplasms but also for a wide range of other diseases.

Covariates used for analysis were chosen based on findings in our previous study of the entire vaccinated VHA population.^4^ Therefore, demographic characteristics included age and biologic sex but not US region nor self-reported race or ethnicity. Four time periods for infection were defined based on changes in the predominant viral variant in the US: pre-Delta (before July 1, 2021), Delta (July 1 through December 15, 2021), Omicron A (December 16, 2021, through February 28, 2022, when the BA.1 and BA.2 variants predominated), and Omicron B (March 1, 2022, through September 30, 2022, when the BA.4 and BA.5 variants predominated). Comorbidities used for multivariable modeling were limited to 6 conditions that were relatively common in the studied population, showed higher prevalence in patients with severe COVID-19, and have been associated with severe COVID-19 in multiple previous studies: Alzheimer disease or dementia, chronic kidney disease, chronic obstructive pulmonary disease, diabetes, heart failure, and peripheral vascular disease. Comorbidities were defined using *ICD-10* codes per the Chronic Conditions Warehouse,^14^ requiring use during the 12-month period before initiation of the primary vaccination series, rather than calendar time, to use the same amount of surveillance time for each patient. Timing relative to vaccination was used to avoid identifying conditions that arose as a consequence of infection.

Receipt of an additional dose or booster dose of any vaccine after completing the initial 2-dose series and before infection was regarded as *boosting*, rather than attempting to define in advance the patients for whom this would be considered completion of initial vaccination by public health authorities. Boosting was modeled as a dichotomous variable without attempting to ascertain receipt of additional doses of vaccines. Time since initial vaccination and time since boosting were not modeled, since these had not been associated with change in risk of severe disease among patients who are immunocompromised.^4^ For treatment with antiviral drugs to reduce risk of progression from mild to moderate infection to severe COVID-19, prescription of any of the monoclonal antibodies (MAB) previously approved was modeled as a single variable, and prescription of an oral antiviral (nirmatrelvir plus ritonavir or molnupiravir) was modeled as a separate single variable. Remdesivir was not included in models, since use to prevent severe COVID-19 (of interest for this study) could not be distinguished from use to treat severe COVID-19.

### Statistical Analysis

Summary data for cases (patients with severe COVID-19) and controls (patients with nonsevere SARS-CoV-2 infection) were compiled as means and SDs (for age and comorbidity score) or as proportions (for all other variables). Multivariable logistic regression was used to estimate the association of demographic and clinical variables with the outcome of severe vs nonsevere SARS-CoV-2 infection. In the full study population, comorbidities were modeled either individually or as a score of 0 to 6 based on the count for each patient. In the 8 individual disease subcohorts, comorbidities were modeled using only the 0 to 6 score. Variables were excluded from analyses by necessity if no patients with that descriptor (eg, antiviral treatment) had severe disease.

*P* values were 2-sided, and statistical significance was set at *P* = .05. All analyses were performed in R software version 4.0.3 (R Project for Statistical Computing) and SAS version 9.4 (SAS Institute). Data were analyzed from July 28 to December 30, 2023.

## Results

**Figure 1.**
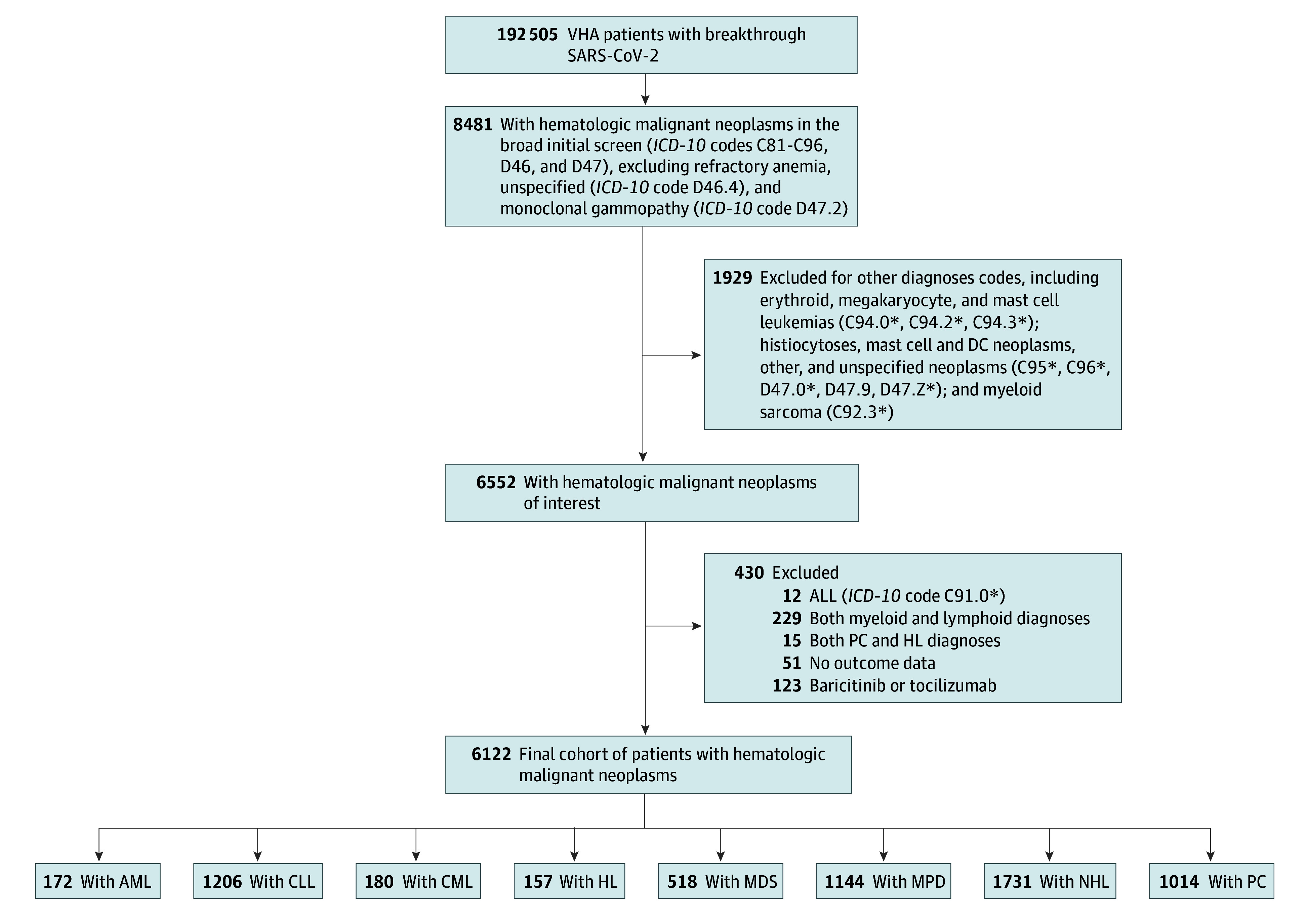
Assembly of the Cohort and Subcohorts of Patients With Hematologic Malignant Neoplasms The source population was all Veteran Health Administration (VHA) patients with documented microbiologically confirmed SARS-CoV-2 infection at any time after vaccination through September 30, 2022. ALL indicates acute lymphocytic leukemia; AML, acute myeloblastic leukemia; CLL, chronic lymphocytic leukemia; CML, chronic myelogenous leukemia (code indicating positive for breakpoint cluster region-ABL); HL, Hodgkin lymphoma; *ICD-10*, *International Statistical Classification of Diseases and Related Health Problems, Tenth Revision;* MDS, myelodysplastic syndromes; MPD, myeloproliferative disorders (not meeting criteria for MDS, CML, or AML); NHL, non-Hodgkin lymphoma; and PC, plasmacytic malignant neoplasms.

We identified 6122 previously vaccinated patients (5844 [95.5%] male; mean [SD] age, 70.89 [11.57] years) with hematologic cancers who developed microbiologically confirmed SARS-CoV-2 infection between January 1, 2021, and September 30, 2022. Of these, 4108 patients were diagnosed with lymphoid malignant neoplasms (157 patients with HL; 1731 patients with NHL; 1206 patients with CLL; 1014 patients with PC) and 2014 patients were diagnosed with myeloid malignant neoplasms (1144 patients with MPD; 518 patients with MDS; 180 patients with CML; 172 patients with AML), with 430 patients (including all 12 patients with acute lymphocytic leukemia) meeting exclusion criteria (Figure 1). Demographic and clinical characteristics of these groups are shown in Table 1 and eTables 2 through 9 in Supplement 1, stratified by severe and nonsevere COVID-19.

**Table 1.** Clinical and Demographic Characteristics for the Full Cohort of Patients With Hematologic Malignant Neoplasms and SARS-CoV-2 Infection After Vaccination, Stratified by Severity

Characteristic	SARS-CoV-2 severity, No. (%)		Group-level proportion with severe disease, %
	Nonsevere (n = 4821)	Severe (n = 1301)	
Medications			
Glucocorticoids	2753 (57.1)	877 (67.4)	24.2
BTK inhibitor	296 (6.1)	120 (9.2)	28.8
Venetoclax	137 (2.8)	45 (3.5)	24.7
Thalidomide, lenalidomide, or pomalidomide	405 (8.4)	151 (11.6)	27.2
Proteosome inhibitor	286 (5.9)	127 (9.8)	30.8
Belinostat	1 (<0.1)	0	NA
MAB for myeloma	142 (3.0)	66 (5.1)	31.7
Hydroxyurea	284 (5.9)	66 (5.1)	18.9
Methotrexate	172 (3.6)	61 (4.7)	26.2
JAK2 inhibitor	58 (1.2)	20 (1.5)	25.6
BCR-ABL inhibitor	131 (2.7)	22 (1.7)	14.4
Lymphocyte-depleting	594 (12.3)	226 (17.4)	27.6
Cytotoxic chemotherapy	837 (17.4)	316 (24.3)	27.4
Immunosuppressing nonhematologic	208 (4.3)	66 (5.1)	24.1
Treatment group			
Both GC and non-GC	1386 (28.8)	519 (39.9)	27.2
GC only	1367 (28.4)	358 (27.5)	20.8
Non-GC only	722 (15.0)	182 (14.0)	20.1
No medication	1346 (27.9)	242 (18.6)	15.2
Disease group			
Lymphoid	3219 (66.8)	889 (68.3)	21.6
Myeloid	1602 (33.2)	412 (31.7)	20.5
Disease class			
AML	129 (2.7)	43 (3.3)	25.0
CLL	931 (19.3)	275 (21.1)	22.8
CML	152 (3.2)	28 (2.2)	15.6
HL	138 (2.9)	19 (1.5)	12.1
MDS	372 (7.7)	146 (11.2)	28.2
MPD	949 (19.7)	195 (15.0)	17.0
NHL	1370 (28.4)	361 (27.8)	20.9
PC	780 (16.2)	234 (18.0)	23.1
Age, mean (SD), y	69.69 (11.98)	75.33 (10.06)	NA
Sex			
Female	263 (5.5)	45 (3.5)	14.6
Male	4558 (94.5)	1256 (96.5)	21.6
Race^a^			
American Indian or Alaska Native	26 (0.5)	8 (0.6)	23.5
Asian	31 (0.6)	5 (0.4)	13.9
Black or African American	1035 (21.5)	264 (20.3)	20.3
Native Hawaiian or Other Pacific Islander	41 (0.9)	7 (0.5)	14.6
White	3374 (70.0)	948 (72.9)	21.9
Unknown	314 (6.5)	69 (5.3)	18.0
Ethnicity^a^			
Hispanic or Latino	387 (8.0)	109 (8.4)	22.0
Not Hispanic or Latino	4255 (88.3)	1147 (88.2)	21.2
Unknown	179 (3.7)	45 (3.5)	20.1
Period of dominant variant			
Delta	771 (16.0)	331 (25.4)	30.0
Omicron A^b^	1923 (39.9)	446 (34.3)	18.8
Omicron B^c^	2005 (41.6)	475 (36.5)	19.2
Pre-Delta	122 (2.5)	49 (3.8)	28.7
Vaccine Type			
Ad26.COV2.S	256 (5.3)	76 (5.8)	22.9
mRNA-1273	2259 (46.9)	562 (43.2)	19.9
BNT162b2	2306 (47.8)	663 (51.0)	22.3
Booster or additional dose			
Yes	2819 (58.5)	649 (49.9)	18.7
No	2002 (41.5)	652 (50.1)	24.6
Infection before vaccine	221 (4.6)	42 (3.2)	16.0
Comorbidities			
Alzheimer disease or dementia	264 (5.5)	173 (13.3)	39.6
Chronic kidney disease	1332 (27.6)	525 (40.4)	28.3
COPD or bronchiectasis	759 (15.7)	369 (28.4)	32.7
Diabetes	1743 (36.2)	610 (46.9)	25.9
Heart failure	495 (10.3)	297 (22.8)	37.5
Liver or cirrhosis	409 (8.5)	131 (10.1)	24.3
Mobility impairments	78 (1.6)	49 (3.8)	38.6
MS or transverse myelitis	30 (0.6)	5 (0.4)	14.3
Peripheral vascular disease	349 (7.2)	182 (14.0)	34.3
Pressure or chronic ulcers	157 (3.3)	100 (7.7)	38.9
Schizophrenia or psychosis	112 (2.3)	38 (2.9)	25.3
Comorbidity score, mean (SD)^d^	1.03 (1.14)	1.66 (1.39)	NA
Treatment for SARS-CoV-2			
Oral antiviral			
Any	630 (13.1)	25 (1.9)	3.8
Nirmatrelvir plus ritonavir	450 (9.3)	17 (1.3)	3.6
Molnupiravir	181 (3.8)	8 (0.6)	4.2
MAB	558 (11.6)	83 (6.4)	12.9
No antiviral	3667 (76.1)	1197 (92.0)	24.6

Abbreviations: AML, acute myeloblastic leukemia; BCR, breakpoint cluster region; BTK, Bruton tyrosine kinase; CLL, chronic lymphocytic leukemia; CML, chronic lymphocytic leukemia; COPD, chronic obstructive pulmonary disease; GC, glucocorticoid; HL, Hodgkin lymphoma; MAB, monoclonal antibodies; MDS, myelodysplastic syndromes; MPD, myeloproliferative disorder without a diagnosis of MDS or CML; MS, multiple sclerosis; NA, not applicable; NHL, non-Hodgkin lymphoma; PC, plasmacytoid malignancy (myeloma or Waldenstrom).

^a^ Race and ethnicity are self-reported by patients at the time of registration, with Hispanic or Latino ethnicity a separate category from race.

^b^ Defined as infection between December 16, 2021, and February 28, 2022.

^c^ Defined as infection between March 1 and September 30, 2022.

^d^ Range, 0 to 6, with higher score indicating more comorbidities.

In the full study population, 1301 patients (21.3%) had severe COVID-19, including 889 patients (21.6%) in the lymphoid group and 412 patients (20.5%) in the myeloid group (Table 1). Proportions of patients with severe COVID-19 were higher among those who had been recently treated with nonglucocorticoid antineoplastic or immune-suppressive drugs and/or glucocorticoids (1059 patients [23.4%]) than those who were not recently treated (242 patients [15.2%]) (Table 1). Proportions varied from 19 patients with HL (12.1%) to 146 patients with MDS (28.2%), but age and comorbidities also differed markedly among the disease groups (eTable 10 and eTable 11 in Supplement 1). Other groups with comparatively low proportions of severe disease were NHL (62 patients [13.7%]) or MPD (48 patients [13.9%]) without recent treatment and CML either without treatment or receiving breakpoint cluster region (BCR)–ABL inhibitors (19 patients [12.7%]), ie, the subgroups least likely to be immunocompromised. Data on the timing of vaccination relative to infection are shown in eAppendix in Supplement 1.

**Figure 2.**
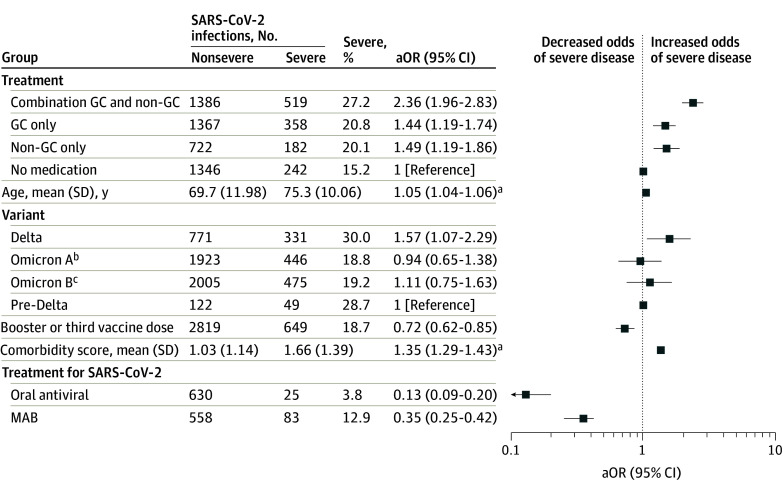
Demographic and Clinical Variables Associated With Severe COVID-19 After Vaccination in Patients With Hematologic Malignant Neoplasms Adjusted odds ratios (aOR) of multivariable logistic regression are shown. Comorbidity score ranged from 0 to 6 and included Alzheimer disease or dementia, chronic kidney disease, chronic obstructive pulmonary disorder, diabetes, heart failure, and peripheral vascular disease. GC indicates glucocorticoid; MAB, monoclonal antibodies targeting SARS-CoV-2. ^a^aORs are presented per 1-unit increase. ^b^Defined as infection between December 16, 2021, and February 28, 2022. ^c^Defined as infection between March 1 and September 30, 2022.

In multivariable logistic regression analysis including all patients, treatment prior to infection with either nonglucocorticoid antineoplastic or immune-suppressive drugs alone (aOR, 1.50; 95% CI, 1.19-1.87) or glucocorticoids alone (aOR, 1.38; 95% CI, 1.14-1.68) was associated with increased odds of severe disease compared with no treatment, and treatment with both glucocorticoids and nonglucocorticoids was associated with even greater odds of severe disease (aOR, 2.32; 95% CI, 1.93-2.80) (Table 2 and Figure 2). Within the subtypes of malignant neoplasms, antineoplastic or immune-suppressive treatment was significantly associated with increased odds of severe disease only among patients with NHL, CLL, PC, or MDS (eTables 2-9 in Supplement 1). However, this result is difficult to interpret due to varying frequencies of untreated patients in the 8 different subgroups (6.1%-36.9%) and the differences among drugs commonly used, although use of systemic glucocorticoids was common in all subgroups (48.8%-66.2%) (eTable 10 and eTable 11 in Supplement 1).

**Table 2.** Multivariable Logistic Regression Analyses With Severe vs Nonsevere Infection as the Outcome^a^

Characteristic	Individual comorbidities		Comorbidity score	
	aOR (95% CI)	*P* value	aOR (95% CI)	*P* value
Treatment group				
Combination GC and non-GC	2.32 (1.93-2.80)	<.001	2.36 (1.96-2.83)	<.001
GC only	1.38 (1.14-1.68)	.24	1.44 (1.19-1.74)	.05
Non-GC only	1.50 (1.19-1.87)	.88	1.49 (1.19-1.86)	.09
No medication	1 [Reference]	NA	1 [Reference]	NA
Age, per 1-y increase	1.05 (1.04-1.06)	<.001	1.05 (1.04-1.06)	<.001
Sex				
Female	1.23 (0.87-1.75)	.24	1.22 (0.86-1.71)	.27
Male	1 [Reference]	NA	1 [Reference]	NA
Period of dominant variant				
Delta	1.57 (1.07-2.29)	<.001	1.57 (1.07-2.29)	<.001
Omicron A^b^	0.94 (0.64-1.37)	.007	0.94 (0.65-1.38)	.007
Omicron B^c^	1.09 (0.74-1.61)	.69	1.11 (0.75-1.63)	.77
Pre-Delta	1 [Reference]	NA	1 [Reference]	NA
Booster or additional dose				
Yes	0.73 (0.62-0.86)	<.001	0.72 (0.62-0.85)	<.001
No	1 [Reference]	NA	1 [Reference]	NA
Comorbidities				
Alzheimer disease or dementia	1.55 (1.24-1.95)	<.001	NR	NR
Chronic kidney disease	1.14 (0.98-1.33)	.08	NR	NR
COPD or bronchiectasis	1.54 (1.31-1.81)	<.001	NR	NR
Diabetes	1.28 (1.11-1.47)	<.001	NR	NR
Heart failure	1.61 (1.34-1.93)	<.001	NR	NR
Peripheral vascular disease	1.31 (1.06-1.62)	.01	NR	NR
Comorbidity score, per 1-unit increase^d^	NR	NR	1.35 (1.29-1.43)	<.001
Treatment for SARS-CoV-2				
Oral antiviral	0.13 (0.09-0.20)	<.001	0.13 (0.09-0.20)	<.001
MAB	0.33 (0.25-0.42)	<.001	0.35 (0.25-0.42)	<.001
No antiviral	1 [Reference]	NA	1 [Reference]	NA

Abbreviations: aOR, adjusted odds ratio; COPD, chronic obstructive pulmonary disease; GC, glucocorticoid; MAB, monoclonal antibodies; NA, not applicable; NR, not reported.

^a^ aORs, 95% CIs, and *P* values are shown for all variables that were included in the multivariable models, with unmodeled variables denoted as NR.

^b^ Defined as infection between December 16, 2021, and February 28, 2022.

^c^ Defined as infection between March 1 and September 30, 2022.

^d^ Range, 0 to 6, with higher score indicating more comorbidities.

Age was associated with increased odds of severe disease in the full study population (aOR per 1-year increase, 1.05; 95% CI, 1.04-1.06) and all subtypes except HL (Table 2; eTables 2-9 in Supplement 1). Among comorbidities, Alzheimer disease or dementia, chronic obstructive pulmonary disease, diabetes, heart failure, and peripheral vascular disease were associated with increased odds of severe disease, and odds of severe COVID-19 increased in patients with multiple comorbidities (aOR per comorbidity, 1.35; 95% CI, 1.29-1.43) (Table 2). The comorbidity score was also associated with increased odds of severe disease in all lymphoid malignant neoplasms groups and MPD (eTables 2-9 in Supplement 1).

A third or booster vaccine dose was documented in 3468 patients (56.6%) overall, ranging from 43.9% to 61.4% of patients across subgroups. Compared with patients with no documented booster, patients who received a booster dose had reduced odds of severe COVID-19 in the full cohort (aOR, 0.73; 95% CI, 0.62-0.86) (Table 2), but this observation typically did not reach statistical significance in subgroups (eTables 2-9 in Supplement 1).

Overall, 1258 patients (20.5%) received an oral antiviral drug or MAB, and of these, 104 patients (8.3%) developed severe COVID-19 (Table 1). Severe disease was seen in 1197 of 4864 patients (24.6%) without such treatment. However, interpreting the association of antiviral treatment is confounded by the contraindication for use in patients who already have severe disease at initial evaluation, so the very low aOR (Table 2; eTables 2-9 in Supplement 1) should be interpreted with caution. Because use of oral antivirals was restricted to the Omicron B period, we performed an additional analysis limited to infections during that period (eAppendix, eTable 12, and eTable 13 in Supplement 1).

Among all patients, 214 (3.5%) died within 28 days of the positive SARS-CoV-2 test result (eTable 14 in Supplement 1). The numbers were too small to analyze, and health record review was not performed to determine whether COVID-19 was a contributor to death. With those caveats, the data were most notable for the low rates of death in patients who received oral antivirals (3 patients [0.5%]) or MABs (6 patients [0.9%]).

## Discussion

In this case-control study among VHA patients with history of hematologic cancers who were diagnosed with SARS-CoV-2 infection after vaccination, a large proportion developed severe COVID-19 (21.3%). Age and 6 specific common comorbidities were associated with odds of severe disease, as in the general population, with a 6- to 7-year increase in age having a similar aOR (approximately 1.33-1.38) as a comorbidity (1.35).

Among patients in subsets likely to have been cured or to have nonsevere hematologic disease (eg, HL, NHL, CLL, PC, or MPD without recent treatment), 12% to 15% developed severe COVID-19, which provides a reasonable estimate of baseline risk conferred by age and comorbidities in the population studied. Adding an active malignant neoplasm and its treatment to this baseline, it was not surprising that the proportion of patients with severe disease remained high in a population in which more than 50% of patients had received at least 1 booster vaccine dose. In contrast, findings with antiviral treatment were encouraging, indicating that use should be promoted and facilitated in patients with hematologic malignant neoplasms. Since only 26.1% of patients with infection after February 2022 had a record of having received antivirals, barely higher than in the VA population as a whole,^15^ approaches to improve uptake of early antiviral treatment should be investigated and implemented.

Our results are consistent with previous literature, which has uniformly shown increased incidence of poor SARS-CoV-2 outcomes in patients with cancer, and specifically hematologic cancer.^2,5,6,7,8,9,10,11,12,13^ We focused on severe vs nonsevere COVID-19 among vaccinated patients with hematologic malignant neoplasms and SARS-CoV-2 infection because comparisons with unvaccinated patients or patients without cancer have been sufficiently investigated and comparison with patients without evidence of infection would have high risk of bias due to differential surveillance and missing data. Even more so than at earlier points in the pandemic, severe and fatal COVID-19 are the outcomes of greatest interest, and studies that have included comparisons with controls without hematologic cancers have typically shown larger differences in rates of severe disease or death than in rates of disease independent of severity.^2,7,10,11,12,16^

Our findings extend and are consistent with the limited observational literature on the benefit of nirmatrelvir plus ritonavir in patients with hematologic malignant neoplasms. Absolute percentages of patients with severe COVID-19 have varied from 2.2% to 5.2% in 3 studies with a total of 547 patients treated with nirmatrelvir plus ritonavir.^17,18,19^ Although precise estimation of the benefit of antiviral therapy would require a rigorous design focused on testing that hypothesis (eg, target trial emulation), the relatively low percentage of patients with progression to severe COVID-19 seen in this study is sufficient to promote more widespread use of early antiviral treatment. Although our finding that patients with MDS had the highest proportion of severe breakthrough COVID-19 was surprising and likely associated with older mean age and more comorbidities, there is precedent for patients with MDS having poor responses to SARS-CoV-2 vaccination, even among those not being treated with immune-suppressive drugs.^20^

Use of antineoplastic or immune-suppressive medications prior to infection, especially use of both systemic glucocorticoids and nonglucocorticoids by the same patient, was associated with increased odds of severe COVID-19. Whether treatment confers risk or is a surrogate marker of an immune system compromised by more severe hematologic disease, or both, is unclear. This problem would be intractable in most observational studies of infections in patients with hematologic malignant neoplasms, but we propose that determining risk among a specific group, like patients with CLL severe enough to require treatment, is more relevant clinically than is dissecting the mechanisms of risk.

### Limitations

This study has some limitations. The VHA population is predominantly male, older, and underrepresented by Hispanic or Latino and Asian patients relative to the general US adult population, so results may not be generalizable to other demographic groups. Our case definitions for hematologic malignant neoplasms were based on *ICD-10* codes and were not validated within the study by health record review. Requiring use of these codes within the data collection period (starting January 1, 2020) should improve positive predictive value for recently active malignant neoplasms compared with scanning over many years, especially for conditions that are curable (eg, HL and some NHL) or for which the diagnosis might have been suspected but not subsequently confirmed (eg, MPD or MDS), but the possibility of misclassification among the untreated patients remains a limitation. Although limitation of the study population to patients recently treated for hematologic malignant neoplasms would improve the positive predictive value, doing so would have prevented estimation of the association of treatment either directly or as a surrogate marker of severe malignant neoplasm. Our description of the subset of patients with good evidence of an active hematologic malignant neoplasm (based on use of nonglucocorticoid immune-suppressive or antineoplastic drugs), receipt of a booster vaccine, and infection after February 2022, gives an estimate of the risk still faced by patients in the absence of antiviral treatment.

Several limitations are related to medications. Evaluating the associations of specific immune-suppressive drugs would be challenging. In addition to confounding by indication, multiple drugs are unique to different diseases (eg, Bruton tyrosine kinase inhibitors in CLL, BCR-ABL inhibitors in CML, and multiple drugs in myeloma) and vary from highly to not immunosuppressive, and the frequency of use was too low to compare drugs within a given disease. Use of these drugs was highly enriched in the appropriate diseases, supporting the *ICD-10*–based approach that we used. We excluded dexamethasone from the list of immune-suppressive drugs screened for use prior to breakthrough infection, out of concern that early use to treat severe COVID-19 could be misclassified as use prior to infection; the missing data most likely to result from this decision is use of dexamethasone to treat myeloma. However, misclassification of patients as untreated with glucocorticoids for malignant neoplasms or as untreated with an antiviral for nonsevere infection would attenuate the associations observed.

Assessment of comorbidities using *ICD-10*–based algorithms also risks both false-positive and false-negative results that vary with the arbitrary time period being screened for use. However, such errors are likely to be balanced among the groups being compared, since the presence of data on vaccination, SARS-CoV-2 testing, and malignant neoplasms indicate that patients with nonsevere disease were regular users of VHA health care. The variable length of follow-up between vaccination and infection means that missing data on recent-onset comorbidities may be greater among the patients with longer intervals, but we felt it was a priority to avoid inclusion of comorbidities that were caused by SARS-CoV-2 infection.

There remains a risk of use of non-VHA care leading to missing data as a source of additional limitations. Use of home antigen testing for SARS-CoV-2 may mean that mild infections were not documented in the VHA electronic health record and therefore were omitted, and the effect would be to overestimate the frequency of severe disease. Use of non-VHA care is also an important potential source of missing data on hospitalizations (a component of our definition of severe COVID-19), but hospitalizations are captured for approximately 70% of non-VHA hospitals that contract for reimbursement. Because providing third or booster vaccine doses at commercial pharmacies became common starting in mid-2021, we did not attempt to ascertain receipt of multiple additional vaccine doses, and some patients may have been misclassified as not having received any additional dose. However, receipt of multiple doses is expected to be higher in patients with cancer than in the general population, so it is likely that patients who received multiple additional doses remain at risk of severe COVID-19.

Oral antivirals and MABs may also have been received outside the VHA, and use of remdesivir for nonsevere disease could not be distinguished from use for severe disease.^15^ Missed documentation of either additional vaccine doses or antiviral treatment would reduce the estimated magnitude of benefit. In contrast, initial presentation with severe disease, as a contraindication for oral antiviral or MAB therapy, would magnify the apparent benefit of antivirals. The net result is that the estimated aORs for antivirals are imprecise, but the absolute difference in proportions of severe disease among treated and untreated patients in the Omicron B period is sufficiently large to conclude that antivirals were associated with benefit and that strategies for increased use should be implemented.

A comprehensive analysis of patients with hematologic cancers would require measurement of multiple laboratory tests over time, which was outside the scope of this study. In creating cancer-type subgroups, such as NHL and PC, we lost the most granular detail about the type of cancer to have subgroups sufficiently large to interpret. In the hope of providing the medical community with as much useful data as possible, we have assembled summary tables even where analysis was inconclusive.

## Conclusions

These findings suggest that patients receiving antineoplastic or immune-suppressive treatment for hematologic malignant neoplasms remain at high risk for severe COVID-19 in the event of infection with SARS-CoV-2 after vaccination. Age and comorbidities were associated with increased odds of severe disease, with aORs similar to those for the general VHA population. Although the magnitude of benefit of antiviral treatments during nonsevere disease could not be quantified, the relatively low proportion of treated patients who developed severe COVID-19 is sufficient to promote greater use.

## Notes

Supplement 1. **eTable 1.** Data Dictionary **eTable 2.** Patients With Hodgkin Lymphoma: Data and Analysis **eTable 3.** Patients With Non-Hodgkin Lymphoma: Data and Analysis **eTable 4.** Patients With Chronic Lymphocytic Leukemia: Data and Analysis **eTable 5.** Patients With Plasmacytoid Malignant Neoplasms: Data and Analysis **eTable 6.** Patients With Myeloproliferative Disorders: Data and Analysis **eTable 7.** Patients With Myelodysplastic Syndromes: Data and Analysis **eTable 8.** Patients With Chronic Myelogenous Leukemia: Data and Analysis **eTable 9.** Patients With Acute Myeloblastic Leukemia: Data and Analysis **eTable 10.** Comparison Across Classes of Hematologic Malignant Neoplasm: All Patients **eTable 11.** Comparison Across Classes of Hematologic Malignant Neoplasm: Patients With Severe COVID-19 **eAppendix.** Timing of Vaccination and Infection: Subcohorts of Patients Infected in Omicron B Period **eTable 12.** Patients With Infection Between March 1, 2022, and September 30, 2022: Data and Analysis **eTable 13.** Patients With Severe COVID-19, Stratified by Age and Number of Comorbidities **eTable 14.** Patients Who Died Within 28 Days of Breakthrough Infection: Data Summary

Supplement 2.
Data Sharing Statement
