# Supplementary material for: Severe COVID-19 in Vaccinated Adults With Hematologic Cancers in the Veterans Health Administration
Source: JAMA Netw Open. 2024 Feb 23;7(2):e240288. doi: 10.1001/jamanetworkopen.2024.0288 (PMC10891464; doi:10.1001/jamanetworkopen.2024.0288)
Supplement: Supplement 1. — eTable 1. Data Dictionary eTable 2. Patients With Hodgkin Lymphoma: Data and Analysis eTable 3. Patients With Non-Hodgkin Lymphoma: Data and Analysis eTable 4. Patients With Chronic Lymphocytic Leukemia: Data and Analysis eTable 5. Patients With Plasmacytoid Malignant Neoplasms: Data and Analysis eTable 6. Patients With Myeloproliferative Disorders: Data and Analysis eTable 7. Patients With Myelodysplastic Syndromes: Data and Analysis eTable 8. Patients With Chronic Myelogenous Leukemia: Data and Analysis eTable 9. Patients With Acute Myeloblastic Leukemia: Data and Analysis eTable 10. Comparison Across Classes of Hematologic Malignant Neoplasm: All Patients eTable 11. Comparison Across Classes of Hematologic Malignant Neoplasm: Patients With Severe COVID-19 eAppendix. Timing of Vaccination and Infection: Subcohorts of Patients Infected in Omicron B Period eTable 12. Patients With Infection Between March 1, 2022, and September 30, 2022: Data and Analysis eTable 13. Patients With Severe COVID-19, Stratified by Age and Number of Comorbidities eTable 14. Patients Who Died Within 28 Days of Breakthrough Infection: Data Summary [file jamanetwopen-e240288-s001.pdf]

Supplemental Online Content

Anand ST, Vo AD, La J, et al. Severe COVID-19 in vaccinated adults with hematologic malignant neoplasms in the Veterans Health Administration. *JAMA Netw Open*. 2024;7(2):e240288. doi:10.1001/jamanetworkopen.2024.0288

eTable 1. Data Dictionary

eTable 2. Patients With Hodgkin Lymphoma: Data and Analysis

eTable 3. Patients With Non-Hodgkin Lymphoma: Data and Analysis

eTable 4. Patients With Chronic Lymphocytic Leukemia: Data and Analysis

eTable 5. Patients With Plasmacytoid Malignant Neoplasms: Data and Analysis

eTable 6. Patients With Myeloproliferative Disorders: Data and Analysis

eTable 7. Patients With Myelodysplastic Syndromes: Data and Analysis

eTable 8. Patients With Chronic Myelogenous Leukemia: Data and Analysis

eTable 9. Patients With Acute Myeloblastic Leukemia: Data and Analysis

eTable 10. Comparison Across Classes of Hematologic Malignant Neoplasm: All Patients

eTable 11. Comparison Across Classes of Hematologic Malignant Neoplasm: Patients With Severe COVID-19

eAppendix. Timing of Vaccination and Infection: Subcohorts of Patients Infected in Omicron B Period

eTable 12. Patients With Infection Between March 1, 2022, and September 30, 2022: Data and Analysis

eTable 13. Patients With Severe COVID-19, Stratified by Age and Number of Comorbidities

eTable 14. Patients Who Died Within 28 Days of Breakthrough Infection: Data Summary

This supplemental material has been provided by the authors to give readers additional information about their work.

© 2024 Anand ST et al. *JAMA Network Open.*

eTable 1. Data dictionary

Variable	Definition
Breakthrough infection	Positive SARS-CoV-2 test (PCR or antigen) at least 14 days after the second dose of mRNA vaccine or first dose of adenoviral vaccine. Only the first breakthrough infection was analyzed
Severe	Hospitalized with either hypoxemia, mechanical ventilation or intubation, or use of dexamethasone; or death from any cause 2-28 days after the positive test
Non-severe	Not hospitalized, or hospitalized and not meeting criteria for severe disease
Hospitalization	Inpatient admission (prevalent) to an acute-care service at a VA Medical Center, or admission to a non-VA hospital contracted with the VA.
Mechanical ventilation or intubation	CPT codes 94002, 94003, 94004, 94005, or 31500; or ICD-10 codes Z99.1, Z99.11, Z99.12, or J95.851; or ICD-9 codes 96.7, 96.70, 96.71, 96.72, 96.0, 96.01, 96.02, 96.03, 96.04, or 96.05
Hypoxemia	Minimum SpO2 < 94% or any use of supplemental oxygen, as determined between days –1 and +14 relative to the first positive SARS-CoV-2 test
Dexamethasone	As recorded in structured data in the VA EHR during an acute-care hospitalization
Sex	As recorded in structured data in the VA EHR, self-reported by patients at enrollment in VA care
Age	In years at time of breakthrough infection
Race and Ethnicity	As recorded in structured data in the VA ESR, self-reported by patients at enrollment in VA care. At the time when most patients enrolled, the categories were American Indian or Alaska Native, Asian, Black or African American, Native Hawaiian or Other Pacific Islander, or White, without a category for "other" or mixed/multiple. Hispanic/Latino ethnicity is self-reported separately from race. For either race or ethnicity, "Unknown" indicates that no category was selected by the patient.
Vaccine Type	Defined based on the type of the initial vaccination, as recorded in the VA COVID-19 Shared Data Resource. (Patients with first and second doses of different types were excluded.)
Janssen	Patient considered vaccinated 14 days after 1st dose
Moderna	Patient considered vaccinated 14 days after 2nd dose
Pfizer	Patient considered vaccinated 14 days after 2nd dose
Boosted before breakthrough	The patient was considered boosted before breakthrough if the breakthrough infection occurred at least 7 days after receiving the booster shot
History of infection before vaccinated	Previous positive SARS-CoV-2 test (PCR or antigen) no later than 14 days after the second mRNA vaccine or first adenoviral vaccine.
Variant period	Date of positive test defining breakthrough infection:
Pre-delta	Before 7/1/2021
Delta	Between 7/1/2021 and 12/15/2021, inclusive
Omicron A	Between 12/16/2021 and 2/28/2022, inclusive
Omicron B	Between 3/1/2022 and 9/30/2022, inclusive
Antiviral treatment for SARS-CoV-2 infection	Prescription for monoclonal antibodies (bamlanivimab, etesevimab, casirivimab, imdevimab, sotrovimab, or bebtelovimab) or oral antivirals (nirmatrelvir/ritonavir or molnupiravir). Remdesivir was excluded because it used more frequently to treat than to prevent severe COVID-19.
Hematologic cancer	Use of ICD-10 code between 1/1/2020 and 4/5/2023:
Lymphocytic	
Hodgkin's Lymphoma	C81.*
Non-Hodgkin's Lymphoma	C82.*, C83.*, C84.*, C85.*, C86.*, C88.4, C91.5*, C91.A* (includes follicular and non-follicular B-cell, T/NK-cell, MALT, and Burkitt lymphomas)
Chronic lymphocytic leukemia	C91.1*, C91.3*, C91.4*, C91.6* (includes hairy cell and prolymphocytic B-cell and T-cell leukemias)

© 2024 Anand ST et al. *JAMA Network Open.*

Acute lymphoblastic leukemia	C91.0*
Myeloid	
Myeloproliferative disorders	D45, D47.1, D47.3, D47.4, C94.4* (includes polycythemia vera, essential thrombocythemia, and myelofibrosis)
Myelodysplastic syndromes	C94.6, D46.0, D46.1, D46.2*, D46.9, D46.A, D46.B, D46.C, D46.Z, C92.2*, C93.1* (includes atypical CML and chronic myelomonocytic anemia, excludes D46.4 refractory anemia not otherwise specified)
Chronic myelogenous leukemia	C93.1* (code specifies BCR-ABL+)
Acute myelogenous leukemia	C92.0*, C92.4*, C92.5*, C92.6*, C92.A*, C93.0* (includes acute promyelocytic, myelomonocytic, and monocytic leukemias)
Plasma cell / plasmacytoid	C88.0, C90.* (includes multiple myeloma and Waldenstrom's, excludes monoclonal gammopathy)
Comorbidities	These comorbidities were derived based the diagnosis and procedure codes described in the CMS Chronic Conditions Warehouse algorithms (https://www2.ccwdata.org/web/guest/condition-categories) any time within one year before the date of completing the initial vaccine series.
Alzheimers disease and related disorders or senile dementia	
Chronic kidney disease	
Chronic obstructive pulmonary disease and bronchiectasis	
Diabetes	
Heart failure	
Mobility impairments	
Liver disease / cirrhosis and other	
Multiple sclerosis and transverse myelitis	
Peripheral vascular disease	
Pressure and chronic ulcers	
Schizophrenia and other psychotic disorders	
Comorbidity score (0-6)	A per-patient count of 6 key comorbidities: Alzheimer's/dementia, chronic kidney disease, COPD, diabetes, heart failure, and peripheral vascular disease
Treatment for hematologic cancer or with immune-suppressive drugs including glucocorticoids	Order/dispensation of one of the drugs listed below within 3-12 months (12 months for lymphocyte-depleting, 6 months for cytotoxic chemotherapy, 3 months for all others) before date of breakthrough infection.
Glucocorticoids	Methylprednisolone, prednisone (dexamethasone was excluded from the list because it is also used to treat severe COVID-19; hydrocortisone and budesonide were excluded due to frequent topical or inhaled use; other glucocorticoids that were also excluded are exclusively topical, inhaled, or injected locally)
BTK inhibitor	Acalabrutinib, ibrutinib, zanubrutinib
Venetoclax	Venetoclax
"-lidomide"	Lenalidomide, pomalidomide, thalidomide
Proteosome inhibitor	Bortezomib, carfilzomib, ixazomib
Belinostat	Belinostat
MAB for myeloma	Daratumumab, elotuzumab, isatuximab
Hydroxyurea	Hydroxyurea
Methotrexate	Methotrexate
JAK2 inhibitor	Fedratinib, pacritinib, ruxolitinib

© 2024 Anand ST et al. *JAMA Network Open.*

BCR-ABL inhibitor	Bosutinib, dasatinib, imatinib, nilotinib
Lymphocyte-depleting	Alemtuzumab, obinutuzumab, ocrelizumab, rituximab
Cytotoxic chemotherapy	Arsenic, asparaginase, asparaginase erwinia chrysanthemi, azacytidine, bendamustine, bleomycin, busulfan, cabazitaxel, calaspargase, capecitabine, carboplatin, carmustine, cedazuridine, chlorambucil, cisplatin, cladribine, clofarabine, cyclophosphamide, cytarabine, dacarbazine, dactinomycin, daunorubicin, decitabine, docetaxel, doxorubicin, epirubicin, eribulin, etoposide, fludarabine, gemcitabine hydroxycamptothecin, hydroxyurea, idarubicin, ifosfamide, irinotecan, ixabepilone, lomustine, mechlorethamine, melphalan, mitomycin c, mitoxantrone, nelarabine, oxaliplatin, paclitaxel, pegaspargase, pemetrexed, pralatrexate, procarbazine, temozolomide, thioguanine, thiotepa, topotecan, trabectedine, trifluridine, valrubicin, vinblastine, vincristine, vindesine, vinorelbine (fluorouracil was excluded because it is also used topically)
IS non-hematologic	Abatacept, adalimumab, anakinra, azathioprine, belimumab, benralizumab, brodalumab, canakinumab, certolizumab, dimethyl fumarate, dupilumab, etanercept, fingolimod, golimumab, infliximab, ixekizumab, leflunomide, mepolizumab, mercaptopurine, mycophenolate, mycophenolic, natalizumab, rapamycin, rilonacept, sarilumab, secukinumab, sirolimus, teriflunomide, tofacitinib, upadacitinib, ustekinumab, vedolizumab (cyclosporine and tacrolimus were excluded from the list because they are also used topically; patients who received baricitinib or tocilizumab were identified but then excluded due to probable use to treat severe COVID-19 rather than as chronic immune-suppressive treatment, and very low likelihood of being used as part of cancer treatment)

© 2024 Anand ST et al. *JAMA Network Open.*

eTable 2. Clinical and demographic data for patients with Hodgkin's lymphoma and SARS-CoV-2 infection after vaccination, stratified by severity, and results of multivariable logistic regression analyses with severe versus non-severe infection as the outcome. Percentages of patients with severe disease are not calculated for N < 10 for a given variable.

	Severity			Multivariable model
N = 157	Non-severe (N = 138)	Severe (N = 19)	% Severe (12.1%)	aOR (CI), P
**Medications**				
Glucocorticoids	82 (59.4%)	8 (42.1%)	8.9%	
Methotrexate	3 (2.2%)	0 (0.0%)		
Lymphocyte-depleting	8 (5.8%)	2 (10.5%)	20.0%	
Cytotoxic chemotherapy	26 (18.8%)	4 (21.1%)	13.3%	
IS non-hematologic	8 (5.8%)	1 (5.3%)		
**Treatment group**				
Combination that includes both GC and non-GC	30 (21.7%)	4 (21.1%)	11.8%	0.65 (0.15-2.88), 0.60
Glucocorticoids only	52 (37.7%)	4 (21.1%)	7.1%	0.29 (0.07-1.18), 0.04
Non-glucocorticoids only	7 (5.1%)	2 (10.5%)		3.03 (0.46-19.84), 0.08
No medication	49 (35.5%)	9 (47.4%)	15.6%	Referent
**Age**				
Mean (SD), aOR per year	61.13 (13.09)	63.29 (12.49)		1.01 (0.97-1.06), 0.62
**Gender**				
F	17 (12.3%)	2 (10.5%)	10.5%	1.28 (0.23-7.09), 0.78
M	121 (87.7%)	17 (89.5%)	12.3%	Referent
**Period of dominant variant**				
Delta	23 (16.7%)	4 (21.1%)	14.8%	0.27 (0.02-4.01), 0.50
Omicron A	65 (47.1%)	8 (42.1%)	11.0%	0.28 (0.02-3.58), 0.45
Omicron B	47 (34.1%)	6 (31.6%)	11.3%	0.32 (0.02-4.76), 0.72
Pre-delta	3 (2.2%)	1 (5.3%)		Referent
**Vaccine Type**				
Janssen	7 (5.1%)	3 (15.8%)	30.0%	
Moderna	60 (43.5%)	11 (57.9%)	15.5%	
Pfizer	71 (51.5%)	5 (26.3%)	6.6%	
**Immunity-related**				
Boosted	64 (46.4%)	5 (26.3%)	7.2%	0.30 (0.08-1.08), 0.07

© 2024 Anand ST et al. *JAMA Network Open.*

N = 157	Severity: Non-severe (N = 138)	Severity: Severe (N = 19)	% Severe (12.1%)	Multivariable model: aOR (CI), P
No booster	74 (53.6%)	14 (73.7%)	15.9%	Referent
Infection before vaccinated	5 (3.6%)	1 (5.3%)		
**Comorbidities**				
Alzheimer's / dementia	1 (0.7%)	3 (15.8%)		
Chronic kidney disease	25 (18.1%)	7 (36.8%)	21.9%	
COPD / bronchiectasis	14 (10.1%)	2 (10.5%)	12.5%	
Diabetes	37 (26.8%)	7 (36.8%)	15.9%	
Heart failure	14 (10.1%)	4 (21.1%)	22.2%	
Mobility impairments	4 (2.9%)	1 (5.3%)		
MS / transverse myelitis	2 (1.5%)	1 (5.3%)		
Peripheral vascular disease	3 (2.2%)	0 (0.0%)		
Pressure / chronic ulcers	2 (1.5%)	1 (5.3%)		
Schizophrenia / psychosis	3 (2.2%)	0 (0.0%)		
**Comorbidity Score (0 – 6)**				
Mean (SD), per point	0.68 (1.01)	1.21 (1.18)		1.78 (1.13-2.82), 0.01
**Treatment for SARS-CoV-2**				
Oral antiviral	14 (10.1%)	0 (0.0%)	0.0%	ND
Nirmatrelvir / ritonavir	10 (7.3%)	0 (0.0%)	0.0%	
Molnupiravir	4 (2.9%)	0 (0.0%)		
MAB	14 (10.1%)	2 (10.5%)	12.5%	1.10 (0.18-6.69), 0.92
No antiviral	111 (80.4%)	17 (89.5%)	13.3%	Referent

aOR = adjusted odds ratio. CI = 95% confidence interval. MAB = monoclonal antibodies. IS = immune-suppressive medication. GC = glucocorticoid. COPD = chronic obstructive pulmonary disease. MS = multiple sclerosis.

© 2024 Anand ST et al. *JAMA Network Open.*

eTable 3. Clinical and demographic data for patients with non-Hodgkin's lymphoma and SARS-CoV-2 infection after vaccination, stratified by severity, and results of multivariable logistic regression analyses with severe versus non-severe infection as the outcome. Percentages of patients with severe disease are not calculated for N < 10 for a given variable.

N = 1731	Severity		% Severe (20.9%)	Multivariable model
	Non-severe (N = 1370)	Severe (N = 361)		aOR (CI), P
**Medications**				
Glucocorticoids	880 (64.2%)	266 (73.7%)	23.2%	
BTK inhibitor	25 (1.8%)	7 (1.9%)	21.9%	
Venetoclax	3 (0.2%)	2 (0.6%)		
"-lidomide"	28 (2.0%)	14 (3.9%)	33.3%	
Proteosome inhibitor	2 (0.2%)	1 (0.3%)		
Belinostat	1 (0.1%)	0 (0.0%)		
MAB myeloma	2 (0.2%)	0 (0.0%)		
Hydroxyurea	1 (0.1%)	2 (0.6%)		
Methotrexate	90 (6.6%)	33 (9.1%)	26.8%	
BCR-ABL inhibitor	2 (0.2%)	0 (0.0%)		
Lymphocyte-depleting	374 (27.3%)	142 (39.3%)	27.5%	
Cytotoxic chemotherapy	373 (27.2%)	143 (39.6%)	27.7%	
IS non-hematologic	61 (4.5%)	18 (5.0%)	22.8%	
**Treatment group**				
Combination that includes both GC and non-GC	444 (32.4%)	160 (44.3%)	26.5%	2.58 (1.83-3.64), <0.001
Glucocorticoids only	436 (31.8%)	106 (29.4%)	19.6%	1.47 (1.03-2.12), 0.14
Non-glucocorticoids only	98 (7.2%)	33 (9.1%)	25.2%	2.40 (1.45-3.97), 0.06
No medication	392 (28.6%)	62 (17.2%)	13.7%	Referent
**Age**				
Mean (SD), aOR per year	70.02 (11.66)	75.02 (9.77)		1.04 (1.03-1.06), <0.001
**Sex**				
F	74 (5.4%)	14 (3.9%)	15.9%	1.32 (0.71-2.46), 0.39
M	1296 (94.6%)	347 (96.1%)	25.8%	Referent
**Period of dominant variant**				
Delta	224 (16.4%)	78 (21.6%)	25.8%	0.95 (0.48-1.90), 0.66
Omicron A	539 (39.3%)	126 (34.9%)	18.9%	0.73 (0.37-1.45), 0.11

© 2024 Anand ST et al. *JAMA Network Open.*

N = 1731	Severity		% Severe (20.9%)	Multivariable model
	Non-severe (N = 1370)	Severe (N = 361)		aOR (CI), P
Omicron B	574 (41.9%)	141 (39.1%)	19.7%	0.90 (0.45-1.82), 0.90
Pre-delta	33 (2.4%)	16 (4.4%)	32.7%	Referent
**Vaccine Type**				
Janssen	64 (4.7%)	15 (4.2%)	19.0%	
Moderna	644 (47.0%)	158 (43.8%)	19.7%	
Pfizer	662 (48.3%)	188 (52.1%)	22.1%	
**Immunity-related**				
Boosted	842 (61.5%)	198 (54.9%)	19.0%	0.77 (0.57-1.04), 0.09
No booster	528 (38.5%)	163 (45.1%)	23.6%	Referent
Infection before vaccinated	53 (3.9%)	12 (3.3%)	18.5%	
**Comorbidities**				
Alzheimer's / dementia	56 (4.1%)	39 (10.8%)	41.1%	
Chronic kidney disease	322 (23.5%)	134 (37.1%)	29.4%	
COPD / bronchiectasis	213 (15.6%)	112 (31.0%)	34.5%	
Diabetes	511 (37.3%)	175 (48.5%)	25.5%	
Heart failure	123 (9.0%)	83 (23.%)	40.3%	
Mobility impairments	14 (1.0%)	10 (2.8%)	41.7%	
MS / transverse myelitis	3 (0.2%)	0 (0.0%)		
Peripheral vascular disease	87 (6.4%)	46 (12.7%)	34.6%	
Pressure / chronic ulcers	30 (2.2%)	24 (6.7%)	44.4%	
Schizophrenia / psychosis	29 (2.1%)	7 (1.9%)	19.4%	
**Comorbidity Score (0 – 6)**				
Mean (SD), aOR per point	0.96 (1.08)	1.63 (1.37)		1.42 (1.29-1.57), <0.001
**Treatment for SARS-CoV-2**				
Oral antiviral	199 (14.5%)	10 (2.8%)	4.8%	0.17 (0.08-0.32), <0.001
Nirmatrelvir / ritonavir	154 (11.2%)	7 (1.9%)	4.3%	
Molnupiravir	45 (3.3%)	3 (0.8%)	6.3%	
MAB	145 (10.6%)	28 (7.8%)	16.2%	0.50 (0.32-0.78), 0.003
No antiviral	1036 (75.6%)	323 (89.5%)	23.8%	Referent

aOR = adjusted odds ratio. CI = 95% confidence interval. BTK = Bruton tyrosine kinase. "-lidomide" = thalidomide, lenalidomide, pomalidomide. MAB = monoclonal antibodies. IS = immune-suppressive medication. GC = glucocorticoid. COPD = chronic obstructive pulmonary disease. MS = multiple sclerosis.

© 2024 Anand ST et al. *JAMA Network Open.*

eTable 4. Clinical and demographic data for patients with chronic lymphocytic leukemia and SARS-CoV-2 infection after vaccination, stratified by severity, and results of multivariable logistic regression analyses with severe versus non-severe infection as the outcome. Percentages of patients with severe disease are not calculated for N < 10 for a given variable.

N = 1206	Severity: Non-severe (N = 931)	Severity: Severe (N = 275)	% Severe (22.8%)	Multivariable model: aOR (CI), P
**Medications**				
Glucocorticoids	477 (51.2%)	187 (68.0%)	28.2%	
BTK inhibitor	238 (25.6%)	100 (36.4%)	29.6%	
Venetoclax	71 (7.6%)	20 (7.3%)	22.0%	
“-lidomide”	0 (0.0%)	2 (0.7%)		
MAB for myeloma	1 (0.1%)	0 (0.0%)		
Hydroxyurea	1 (0.1%)	2 (0.7%)		
Methotrexate	22 (2.4%)	15 (5.5%)	40.5%	
JAK2 inhibitor	1 (0.1%)	0 (0.0%)		
BCR-ABL inhibitor	1 (0.1%)	0 (0.0%)		
Lymphocyte-depleting	134 (14.4%)	52 (18.9%)	28.0%	
Cytotoxic chemotherapy	107 (11.5%)	42 (15.3%)	28.2%	
IS non-hematologic	13 (1.4%)	7 (2.6%)	35.0%	
**Treatment group**				
Any medication including glucocorticoid combination	225 (24.2%)	117 (42.6%)	34.2%	3.91 (2.62-5.84), <0.001
Glucocorticoids only	252 (27.1%)	70 (25.5%)	21.7%	1.86 (1.22-2.83), 0.91
Non-glucocorticoids only	144 (15.5%)	34 (12.4%)	19.1%	1.54 (0.94-2.54), 0.30
No medication	310 (33.3%)	54 (19.6%)	14.8%	Referent
**Age**				
Mean (SD), aOR per year	73.35 (8.96)	77.32 (9.06)		1.06 (1.04-1.08), <0.001
**Sex**				
F	16 (1.7%)	8 (2.9%)	33.3%	3.44 (1.24-9.51), 0.02
M	915 (98.3%)	267 (97.1%)	22.6%	Referent
**Period of dominant variant**				
Delta	162 (17.4%)	63 (22.9%)	28.0%	1.37 (0.60-3.12), 0.17
Omicron A	366 (39.3%)	107 (38.9%)	22.6%	1.07 (0.47-2.43), 0.90
Omicron B	378 (40.6%)	94 (34.2%)	19.9%	0.96 (0.41-2.21), 0.42

© 2024 Anand ST et al. *JAMA Network Open.*

N = 1206	Severity: Non-severe (N = 931)	Severity: Severe (N = 275)	% Severe (22.8%)	Multivariable model: aOR (CI), P
Pre-delta	25 (2.7%)	11 (4.0%)	30.6%	Referent
**Vaccine Type**				
Janssen	40 (4.3%)	12 (4.4%)	23.1%	
Moderna	451 (48.4%)	116 (42.2%)	20.5%	
Pfizer	440 (47.3%)	147 (53.5%)	25.0%	
**Immunity-related**				
Boosted	587 (63.1%)	153 (55.6%)	20.7%	0.84 (0.60-1.19), 0.34
No booster	344 (36.9%)	122 (44.4%)	26.2%	Referent
Infection before vaccinated	37 (4.0%)	5 (1.8%)	11.9%	
**Comorbidities**				
Alzheimer's / dementia	47 (5.1%)	34 (12.4%)	42.0%	
Chronic kidney disease	240 (25.8%)	90 (32.7%)	27.2%	
COPD / bronchiectasis	139 (14.9%)	63 (22.9%)	31.2%	
Diabetes	340 (36.5%)	115 (41.8%)	25.3%	
Heart failure	79 (8.5%)	47 (17.1%)	37.3%	
Mobility impairments	8 (0.9%)	4 (1.5%)	33.3%	
MS / transverse myelitis	6 (0.6%)	1 (0.4%)		
Peripheral vascular disease	57 (6.1%)	39 (14.2%)	40.6%	
Pressure / chronic ulcers	26 (2.8%)	14 (5.1%)	35.0%	
Schizophrenia / psychosis	18 (1.9%)	5 (1.8%)	21.7%	
**Comorbidity Score (0 – 6)**				
Mean (SD), aOR per point	0.97 (1.08)	1.41 (1.35)		1.29 (1.14-1.45), <0.001
**Treatment for SARS-CoV-2**				
Oral antiviral	150 (16.1%)	4 (1.5%)	2.6%	0.08 (0.03-0.22), <0.001
Nirmatrelvir / ritonavir	99 (10.6%)	3 (1.1%)	2.9%	
Molnupiravir	51 (5.5%)	1 (0.4%)	1.9%	
MAB	140 (15.0%)	23 (8.4%)	14.1%	0.35 (0.21-0.58), <0.001
No antiviral	653 (70.1%)	249 (90.6%)	27.6%	Referent

aOR = adjusted odds ratio. CI = 95% confidence interval. BTK = Bruton tyrosine kinase. "-lidomide" = thalidomide, lenalidomide, pomalidomide. MAB = monoclonal antibodies. IS = immune-suppressive medication. GC = glucocorticoid. COPD = chronic obstructive pulmonary disease. MS = multiple sclerosis.

© 2024 Anand ST et al. *JAMA Network Open.*

eTable 5. Clinical and demographic data for patients with plasmacytoid malignancies (myeloma or Waldenstrom's) and SARS-CoV-2 infection after vaccination, stratified by severity, and results of multivariable logistic regression analyses with severe versus non-severe infection as the outcome. Percentages of patients with severe disease are not calculated for N < 10 for a given variable.

N = 1014	Severity		% Severe (23.1%)	Multivariable model
	Non-severe (N = 780)	Severe (N = 234)		aOR (CI), P
**Medications**				
Glucocorticoids	447 (57.3%)	147 (62.8%)	24.7%	
BTK inhibitor	32 (4.1%)	13 (5.6%)	28.9%	
Venetoclax	5 (0.6%)	3 (1.3%)		
"-lidomide"	354 (45.4%)	130 (55.6%)	26.9%	
Proteosome inhibitor	284 (36.4%)	125 (53.4%)	30.6%	
MAB myeloma	139 (17.8%)	66 (28.2%)	32.2%	
Methotrexate	20 (2.6%)	2 (0.9%)	9.1%	
BCR-ABL inhibitor	0 (0.0%)	1 (0.4%)		
Lymphocyte-depleting	57 (7.3%)	19 (8.1%)	25.0%	
Cytotoxic chemotherapy	175 (22.4%)	63 (26.9%)	26.5%	
IS non-hematologic	28 (3.6%)	11 (4.7%)	28.2%	
**Treatment group**				
Combination that includes both GC and non-GC	315 (40.4%)	119 (50.9%)	27.4%	3.06 (1.84-5.09), <0.001
Glucocorticoids only	132 (16.9%)	28 (12.0%)	17.5%	1.20 (0.65-2.25), 0.04
Non-glucocorticoids only	178 (22.8%)	60 (25.6%)	25.2%	2.66 (1.54-4.60), 0.007
No medication	155 (19.9%)	27 (11.5%)	14.8%	Referent
**Age**				
Mean (SD), aOR per year	71.77 (9.41)	74.50 (9.33)		1.03 (1.01-1.05), <0.001
**Sex**				
F	29 (3.7%)	4 (1.7%)	12.1%	0.87 (0.28-2.74), 0.81
M	751 (96.3%)	230 (98.3%)	23.4%	Referent
**Period of dominant variant**				
Delta	104 (13.3%)	80 (34.2%)	43.5%	3.26 (1.26-8.48), <0.001
Omicron A	321 (41.2%)	75 (32.1%)	18.9%	1.00 (0.38-2.63), 0.08
Omicron B	336 (43.1%)	71 (30.3%)	17.4%	1.02 (0.38-2.76), 0.14
Pre-delta	19 (2.4%)	8 (3.4%)	29.6%	Referent

© 2024 Anand ST et al. *JAMA Network Open.*

N = 1014	Severity		% Severe (23.1%)	Multivariable model
	Non-severe (N = 780)	Severe (N = 234)		aOR (CI), P
**Vaccine Type**				
Janssen	27 (3.5%)	11 (4.7%)	28.9%	
Moderna	348 (44.6%)	92 (39.3%)	20.9%	
Pfizer	405 (51.9%)	131 (56.0%)	24.4%	
**Immunity-related**				
Boosted	512 (65.6%)	113 (48.3%)	18.1%	0.67 (0.45-1.01), 0.05
No booster	268 (34.4%)	121 (51.7%)	31.1%	Referent
Infection before vaccinated	35 (4.5%)	7 (3.0%)	16.7%	
**Comorbidities**				
Alzheimer's / dementia	47 (6.0%)	24 (10.3%)	33.8%	
Chronic kidney disease	277 (35.5%)	124 (53.0%)	30.9%	
COPD / bronchiectasis	113 (14.5%)	57 (24.4%)	33.5%	
Diabetes	299 (38.3%)	129 (55.1%)	30.1%	
Heart failure	100 (12.8%)	56 (23.9%)	35.9%	
Mobility impairments	11 (1.4%)	9 (3.9%)	45.0%	
MS / transverse myelitis	5 (0.6%)	1 (0.4%)		
Peripheral vascular disease	56 (7.2%)	38 (16.2%)	40.4%	
Pressure / chronic ulcers	28 (3.6%)	19 (8.1%)	40.4%	
Schizophrenia / psychosis	18 (2.3%)	9 (3.9%)	33.3%	
**Comorbidity Score (0 – 6)**				
Mean (SD), aOR per point	1.14 (1.14)	1.83 (1.33)		1.47 (1.29-1.67), <0.001
**Treatment for SARS-CoV-2**				
Oral antiviral	94 (12.1%)	6 (2.6%)	6.0%	0.24 (0.10-0.58), 0.001
Nirmatrelvir / ritonavir	65 (8.3%)	3 (1.3%)	4.4%	
Molnupiravir	29 (3.7%)	3 (1.3%)	9.4%	
MAB	97 (12.4%)	15 (6.4%)	13.4%	0.27 (0.14-0.50), <0.001
No antiviral	592 (75.9%)	215 (91.9%)	26.6%	Referent

aOR = adjusted odds ratio. CI = 95% confidence interval. BTK = Bruton tyrosine kinase. "-lidomide" = thalidomide, lenalidomide, pomalidomide. MAB = monoclonal antibodies. IS = immune-suppressive medication. GC = glucocorticoid. COPD = chronic obstructive pulmonary disease. MS = multiple sclerosis.

© 2024 Anand ST et al. *JAMA Network Open.*

eTable 6. Clinical and demographic data for patients with myeloproliferative disorders (not meeting criteria for myelodysplastic syndromes or chronic myelogenous leukemia) and SARS-CoV-2 infection after vaccination, stratified by severity, and results of multivariable logistic regression analyses with severe versus non-severe infection as the outcome. Percentages of patients with severe disease are not calculated for N < 10 for a given variable.

N = 1144	Severity		% Severe (17.0%)	Multivariable model
	Non-severe (N = 949)	Severe (N = 195)		aOR (CI), P
**Medications**				
Glucocorticoids	516 (54.4%)	128 (65.6%)	19.9%	
"-lidomide"	3 (0.3%)	1 (0.5%)		
Hydroxyurea	220 (23.2%)	33 (16.9%)	13.0%	
Methotrexate	18 (1.9%)	2 (1.0%)	10.0%	
JAK2 inhibitor	30 (3.2%)	8 (4.1%)	21.1%	
BCR-ABL inhibitor	2 (0.2%)	0 (0.0%)		
Lymphocyte-depleting	10 (1.1%)	4 (2.1%)	28.6%	
Cytotoxic chemotherapy	35 (3.7%)	17 (8.7%)	32.7%	
IS non-hematologic	52 (5.5%)	14 (7.2%)	21.2%	
**Treatment group**				
Combination that includes both GC and non-GC	180 (19.0%)	47 (24.1%)	20.7%	1.40 (0.86-2.28), 0.10
Glucocorticoids only	336 (35.4%)	81 (41.5%)	19.4%	1.38 (0.90-2.12), 0.07
Non-glucocorticoids only	135 (14.2%)	19 (9.7%)	12.3%	0.69 (0.37-1.27), 0.03
No medication	298 (31.4%)	48 (24.6%)	13.9%	Referent
**Age**				
Mean (SD), aOR per year	63.68 (13.90)	72.09 (11.76)		1.05 (1.03-1.07), <0.001
**Sex**				
F	102 (10.8%)	9 (4.6%)	8.1%	0.71 (0.34-1.51), 0.37
M	847 (89.3%)	186 (95.4%)	18.0%	Referent
**Period of dominant variant**				
Delta	146 (15.4%)	49 (25.1%)	25.1%	3.83 (1.20-12.28), 0.005
Omicron A	382 (40.3%)	61 (31.3%)	13.8%	1.83 (0.58-5.80), 0.47
Omicron B	393 (41.4%)	81 (41.5%)	17.1%	2.76 (0.86-8.87), 0.17
Pre-delta	28 (3.0%)	4 (2.1%)	12.5%	Referent
**Vaccine Type**				

© 2024 Anand ST et al. *JAMA Network Open.*

N = 1144	Severity		% Severe (17.0%)	Multivariable model
	Non-severe (N = 949)	Severe (N = 195)		aOR (CI), P
Janssen	90 (9.5%)	18 (9.2%)	16.7%	
Moderna	435 (45.8%)	80 (41.0%)	15.5%	
Pfizer	424 (44.7%)	97 (49.7%)	23.0%	
**Immunity-related**				
Boosted	436 (45.9%)	78 (40.0%)	15.2%	0.63 (0.42-0.95), 0.03
No booster	513 (54.1%)	117 (60.0%)	18.6%	Referent
Infection before vaccinated	62 (6.5%)	8 (4.1%)	11.4%	
**Comorbidities**				
Alheimer's / dementia	54 (5.7%)	41 (21.0%)	43.2%	
Chronic kidney disease	246 (25.9%)	81 (41.5%)	24.8%	
COPD / bronchiectasis	160 (16.9%)	74 (38.0%)	31.6%	
Diabetes	304 (32.0%)	91 (46.7%)	23.0%	
Heart failure	78 (8.2%)	51 (26.2%)	39.5%	
Mobility impairments	26 (2.7%)	18 (9.2%)	40.9%	
MS / transverse myelitis	9 (1.0%)	2 (1.0%)	18.2%	
Peripheral vascular disease	90 (9.5%)	25 (12.8%)	21.7%	
Pressure / chronic ulcers	49 (5.2%)	25 (12.8%)	33.8%	
Schizophrenia / psychosis	30 (3.2%)	12 (6.2%)	28.6%	
**Comorbidity Score (0-6)**				
Mean (SD), aOR per point	0.98 (1.14)	1.86 (1.46)		1.46 (1.28-1.65), <0.001
**Treatment for SARS-CoV-2**				
Oral antiviral	92 (9.7%)	2 (1.0%)	2.1%	0.09 (0.02-0.38), 0.001
Nirmatrelvir / ritonavir	70 (7.4%)	2 (1.0%)	2.8%	
Molnupiravir	22 (2.3%)	0 (0.0%)	0.0%	
MAB	76 (8.0%)	7 (3.6%)	8.4%	0.20 (0.09-0.48), <0.001
No antiviral	785 (82.7%)	186 (95.4%)	19.2%	Referent

aOR = adjusted odds ratio. CI = 95% confidence interval. BTK = Bruton tyrosine kinase. "-lidomide" = thalidomide, lenalidomide, pomalidomide. MAB = monoclonal antibodies. IS = immune-suppressive medication. GC = glucocorticoid. COPD = chronic obstructive pulmonary disease. MS = multiple sclerosis.

© 2024 Anand ST et al. *JAMA Network Open.*

eTable 7. Clinical and demographic data for patients with myelodysplastic syndromes and SARS-CoV-2 infection after vaccination, stratified by severity, and results of multivariable logistic regression analyses with severe versus non-severe infection as the outcome. Percentages of patients with severe disease are not calculated for N < 10 for a given variable.

N = 518	Severity: Non-severe (N = 372)	Severity: Severe (N = 146)	% Severe (28.2%)	Multivariable model: aOR (CI), P
**Medications**				
Glucocorticoids	216 (58.1%)	97 (66.4%)	31.0%	
Venetoclax	7 (1.9%)	1 (0.7%)		
"-lidomide"	17 (4.6%)	4 (2.7%)	19.0%	
Proteosome	0 (0.0%)	1 (0.7%)		
Hydroxyurea	33 (8.9%)	17 (11.6%)	34.0%	
Methotrexate	10 (2.7%)	7 (4.8%)	41.2%	
JAK2 inhibitor	18 (4.8%)	8 (5.5%)	30.8%	
BCR-ABL inhibitor	2 (0.5%)	1 (0.7%)		
Lymphocyte-depleting	7 (1.9%)	3 (2.1%)	30.0%	
Cytotoxic chemotherapy	57 (15.3%)	22 (15.1%)	27.8%	
IS non-hematologic	21 (5.7%)	8 (5.5%)	27.6%	
**Treatment group**				
Combination that includes both GC and non-GC	89 (23.9%)	39 (26.7%)	30.5%	1.91 (1.05-3.48), 0.19
Glucocorticoids only	127 (34.1%)	58 (39.7%)	31.4%	1.65 (0.96-2.85), 0.56
Non-glucocorticoids only	46 (12.4%)	16 (11.0%)	25.8%	1.59 (0.76-3.34), 0.81
No medication	110 (29.6%)	33 (22.6%)	23.1%	Referent
**Age**				
Mean (SD), aOR per year	75.22 (10.78)	79.73 (8.41)		1.06 (1.03-1.09), <0.001
**Sex**				
F	11 (3.0%)	4 (2.7%)	26.7%	2.10 (0.56-7.86), 0.27
M	361 (97.0%)	142 (97.3%)	28.2%	Referent
**Period of dominant variant**				
Delta	61 (16.4%)	40 (27.4%)	39.6%	1.15 (0.32-4.09), 0.41
Omicron A	136 (36.6%)	48 (32.9%)	26.1%	0.81 (0.23-2.89), 0.51
Omicron B	168 (45.2%)	53 (36.3%)	24.0%	0.84 (0.23-3.06), 0.62
Pre-delta	7 (1.9%)	5 (3.4%)	41.7%	Referent

© 2024 Anand ST et al. *JAMA Network Open.*

N = 518	Severity		% Severe (28.2%)	Multivariable model
	Non-severe (N = 372)	Severe (N = 146)		aOR (CI), P
**Vaccine Type**				
Janssen	11 (3.0%)	10 (6.9%)	47.6%	
Moderna	195 (52.4%)	72 (49.3%)	27.0%	
Pfizer	166 (44.6%)	64 (43.8%)	27.8%	
**Immunity-related**				
Boosted	222 (59.7%)	65 (44.5%)	22.6%	0.61 (0.37-1.01), 0.05
No booster	150 (40.3%)	81 (55.5%)	35.1%	Referent
Infection before vaccinated	17 (4.6%)	6 (4.1%)	26.1%	
**Comorbidities**				
Alzheimer’s / dementia	45 (12.1%)	25 (17.1%)	35.7%	
Chronic kidney disease	139 (37.4%)	63 (43.2%)	31.2%	
COPD / bronchiectasis	77 (20.7%)	44 (30.1%)	36.4%	
Diabetes	148 (39.8%)	66 (45.2%)	30.8%	
Heart failure	79 (21.2%)	43 (29.5%)	35.2%	
Mobility impairments	11 (3.0%)	5 (3.4%)	31.3%	
MS / transverse myelitis	3 (0.8%)	0 (0.0%)		
Peripheral vascular disease	43 (11.6%)	27 (18.5%)	38.6%	
Pressure / chronic ulcers	16 (4.3%)	14 (9.6%)	46.7%	
Schizophrenia / psychosis	10 (2.7%)	4 (2.7%)	28.6%	
**Comorbidity Score (0 – 6)**				
Mean (SD), per point	1.43 (1.37)	1.84 (1.55)		1.15 (0.99-1.33), 0.07
**Treatment for SARS-CoV-2**				
Oral antiviral	45 (12.1%)	2 (1.4%)	4.3%	0.10 (0.02-0.43), 0.02
Nirmatrelvir / ritonavir	30 (8.1%)	1 (0.7%)	3.2%	
Molnupiravir	15 (4.0%)	1 (0.7%)	6.3%	
MAB	45 (12.1%)	5 (3.4%)	10.0%	0.16 (0.06-0.43), <0.001
No antiviral	284 (76.3%)	139 (95.2%)	32.9%)	Referent

aOR = adjusted odds ratio. CI = 95% confidence interval. BTK = Bruton tyrosine kinase. “-lidomide” = thalidomide, lenalidomide, pomalidomide. MAB = monoclonal antibodies. IS = immune-suppressive medication. GC = glucocorticoid. COPD = chronic obstructive pulmonary disease. MS = multiple sclerosis.

© 2024 Anand ST et al. *JAMA Network Open.*

eTable 8. Clinical and demographic data for patients with chronic myelogenous leukemia (coded as BCR-ABL positive) and SARS-CoV-2 infection after vaccination, stratified by severity, and results of multivariable logistic regression analyses with severe versus non-severe infection as the outcome. Percentages of patients with severe disease are not calculated for N < 10 for a given variable.

	Severity			Multivariable model
N = 180	Non-severe (N = 152)	Severe (N = 28)	% Severe (15.6%)	aOR (CI), P
**Medications**				
Glucocorticoids	74 (48.7%)	21 (75.0%)	22.1%	
Hydroxyurea	14 (9.2%)	5 (17.9%)	26.3%	
Methotrexate	4 (2.6%)	1 (3.6%)		
JAK2 inhibitor	2 (1.3%)	2 (7.1%)		
BCR-ABL inhibitor	121 (79.6%)	18 (64.3%)	12.9%	
Lymphocyte-depleting	0 (0.0%)	2 (7.1%)		
Cytotoxic chemotherapy	7 (4.6%)	5 (17.9%)	41.7%	
IS non-hematologic	4 (2.6%)	4 (14.3%)		
**Treatment group**				
Combination that includes both GC and non-GC	60 (39.5%)	18 (64.3%)	23.1%	3.56 (0.38-33.14), 0.10
Glucocorticoids only	14 (9.2%)	3 (10.7%)	17.6%	2.85 (0.22-36.99), 0.45
Non-glucocorticoids only	68 (44.7%)	6 (21.4%)	8.1%	1.00 (0.10-10.14), 0.23
No medication	10 (6.6%)	1 (3.6%)	9.1%	Referent
**Age**				
Mean (SD), aOR per year	66.92 (11.23)	75.24 (11.06)		1.09 (1.03-1.15), 0.003
**Gender**				
F	6 (4.0%)	2 (7.1%)		3.56 (0.43-29.53), 0.24
M	146 (96.1%)	26 (92.9%)	15.1%	Referent
**Period of dominant variant**				
Delta	27 (17.8%)	7 (25.0%)	20.6%	0.51 (0.05-5.68), 0.68
Omicron A	58 (38.2%)	7 (25.0%)	10.8%	0.20 (0.02-2.14), 0.10
Omicron B	64 (42.1%)	12 (42.9%)	15.8%	0.30 (0.02-3.70), 0.51
Pre-delta	3 (2.0%)	2 (7.1%)		Referent
**Vaccine Type**				
Janssen	9 (5.9%)	2 (7.1%)	18.2%	
Moderna	70 (46.1%)	12 (42.9%)	14.6%	

© 2024 Anand ST et al. *JAMA Network Open.*

N = 180	Severity		% Severe (15.6%)	Multivariable model
	Non-severe (N = 152)	Severe (N = 28)		aOR (CI), P
Pfizer	73 (48.0%)	14 (50.0%)	16.1%	
**Immunity-related**				
Boosted	87 (57.2%)	15 (53.6%)	14.7%	1.16 (0.37-3.65), 0.80
No booster	65 (42.8%)	13 (46.4%)	16.7%	Referent
Infection before vaccinated	3 (2.0%)	1 (3.6%)		
**Comorbidities**				
Alzheimer's / dementia	6 (4.0%)	4 (14.3%)	40.0%	
Chronic kidney disease	47 (30.9%)	12 (42.9%)	20.3%	
COPD / bronchiectasis	23 (15.1%)	8 (28.6%)	25.8%	
Diabetes	60 (39.5%)	11 (39.3%)	15.5%	
Heart failure	11 (7.2%)	7 (25.0%)	38.9%	
Mobility impairments	3 (2.0%)	2 (7.1%)		
MS / transverse myelitis	1 (0.7%)	0 (0.0%)		
Peripheral vascular disease	7 (4.6%)	4 (14.3%)	36.4%	
Pressure / chronic ulcers	2 (1.3%)	3 (10.7%)		
Schizophrenia / psychosis	2 (1.3%)	0 (0.0%)		
**Comorbidity Score, 0 – 6**				
Mean (SD), per point	1.01 (1.17)	1.64 (1.34)		1.26 (0.82-1.94), 0.28
**Treatment for SARS-CoV-2**				
Oral antiviral	17 (11.2%)	0 (0.0%)	0.0%	ND
Nirmatrelvir / ritonavir	10 (6.6%)	0 (0.0%)	0.0%	
Molnupiravir	8 (5.3%)	0 (0.0%)		
MAB	21 (13.8%)	1 (3.6%)	4.5%	0.14 (0.02-1.16), 0.07
No antiviral	116 (76.3%)	27 (96.4%)	18.9%	Referent

aOR = adjusted odds ratio. CI = 95% confidence interval. BTK = Bruton tyrosine kinase. "-lidomide" = thalidomide, lenalidomide, pomalidomide. MAB = monoclonal antibodies. IS = immune-suppressive medication. GC = glucocorticoid. COPD = chronic obstructive pulmonary disease. MS = multiple sclerosis.

© 2024 Anand ST et al. *JAMA Network Open.*

eTable 9. Clinical and demographic data for patients with acute myeloblastic leukemia and SARS-CoV-2 infection after vaccination, stratified by severity, and results of multivariable logistic regression analyses with severe versus non-severe infection as the outcome. Percentages of patients with severe disease are not calculated for N < 10 for a given variable.

	Severity			Multivariable model
N = 172	Non-severe (N = 129)	Severe (N = 43)	% Severe (25.0%)	aOR (CI), P
**Medications**				
Glucocorticoids	61 (47.3%)	23 (53.5%)	27.4%	
BTK inhibitor	1 (0.8%)	0 (0.0%)		
Venetoclax	51 (39.5%)	19 (44.2%)	27.1%	
“-lidomide”	3 (2.3%)	0 (0.0%)		
Hydroxyurea	15 (11.6%)	7 (16.3%)	31.8%	
Methotrexate	5 (3.9%)	1 (2.3%)		
JAK2 inhibitor	7 (5.4%)	2 (4.7%)		
BCR-ABL inhibitor	3 (2.3%)	2 (4.7%)		
Lymphocyte-depleting	4 (3.1%)	2 (4.7%)		
Cytotoxic chemotherapy	57 (44.2%)	20 (46.5%)	26.0%	
IS non-hematologic	21 (16.3%)	3 (7.0%)	12.5%	
**Treatment group**				
Combination that includes both GC and non-GC	43 (33.3%)	15 (34.9%)	25.9%	0.87 (0.27-2.88), 0.99
Glucocorticoids only	18 (14.0%)	8 (18.6%)	30.8%	1.24 (0.31-5.07), 0.40
Non-glucocorticoids only	46 (35.7%)	12 (27.9%)	20.7%	0.53 (0.15-1.84), 0.15
No medication	22 (17.1%)	8 (18.6%)	26.7%	Referent
**Age**				
Mean (SD), aOR per year	67.72 (12.26)	74.90 (7.44)		1.08 (1.04-1.14), <0.001
**Sex**				
F	8 (6.2%)	2 (4.7%)		0.94 (0.14-6.50), 0.95
M	121 (93.8%)	41 (95.4%)	25.3%	Referent
**Period of dominant variant**				
Delta	24 (18.6%)	10 (23.3%)	29.4%	0.98 (0.13-7.35), 0.94
Omicron A	56 (43.4%)	14 (32.6%)	20.0%	0.63 (0.08-4.84), 0.29
Omicron B	45 (34.9%)	17 (39.5%)	27.4%	1.31 (0.16-11.00), 0.47
Pre-delta	4 (3.1%)	2 (4.7%)		Referent

© 2024 Anand ST et al. *JAMA Network Open.*

N = 172	Severity		% Severe (25.0%)	Multivariable model
	Non-severe (N = 129)	Severe (N = 43)		aOR (CI), P
**Vaccine Type**				
Janssen	8 (6.2%)	5 (11.6%)	38.5%	
Moderna	56 (43.4%)	21 (48.8%)	27.3%	
Pfizer	65 (50.4%)	17 (39.5%)	20.7%	
**Immunity-related**				
Boosted	69 (53.5%)	22 (51.2%)	24.2%	1.03 (0.36-2.94), 0.96
No booster	60 (46.5%)	21 (48.8%)	25.9%	
Infection before vaccine	9 (7.0%)	2 (4.7%)	18.2%	
**Comorbidities**				
Alzheimer's / dementia	8 (6.2%)	3 (7.0%)	27.3%	
Chronic kidney disease	36 (27.9%)	14 (32.6%)	28.0%	
COPD / bronchiectasis	20 (15.5%)	9 (20.9%)	31.0%	
Diabetes	44 (34.1%)	16 (37.2%)	26.7%	
Heart failure	11 (8.5%)	6 (14.0%)	35.3%	
Mobility impairments	1 (0.8%)	0 (0.0%)		
MS / transverse myelitis	1 (0.8%)	0 (0.0%)		
Peripheral vascular disease	6 (4.7%)	3 (7.0%)		
Pressure / chronic ulcers	4 (3.1%)	0 (0.0%)		
Schizophrenia / psychosis	2 (1.6%)	1 (2.3%)		
**Comorbidity Score (0 – 6)**				
Mean (SD), aOR per point	0.97 (1.07)	1.19 (1.18)		1.04 (0.71-1.54), 0.83
**Treatment for SARS-CoV-2**				
Oral antiviral	19 (14.7%)	1 (2.3%)	5.0%	0.08 (0.01-0.68), 0.02
Nirmatrelvir / ritonavir	12 (9.3%)	1 (2.3%)	7.7%	
Molnupiravir	7 (5.4%)	0 (0.0%)		
MAB	20 (15.5%)	2 (4.7%)	9.1%	0.18 (0.03-0.92), 0.04
No antiviral	90 (70.0%)	41 (95.4%)	31.3%	Referent

aOR = adjusted odds ratio. CI = 95% confidence interval. BTK = Bruton tyrosine kinase. "-lidomide" = thalidomide, lenalidomide, pomalidomide. MAB = monoclonal antibodies. IS = immune-suppressive medication. GC = glucocorticoid. COPD = chronic obstructive pulmonary disease. MS = multiple sclerosis.

© 2024 Anand ST et al. *JAMA Network Open.*

eTable 10. Clinical characteristics of patients with SARS-CoV-2 infection after vaccination, by subgroup of hematologic malignancy.

	All	HL	NHL	CLL	PC	MPD	MDS	CML	AML
N	6122	157	1731	1206	1014	1144	518	180	172
% severe	21.3%	12.1%	20.9%	22.8%	23.1%	17.0%	28.2%	15.6%	25.0%
**Medications**									
Glucocorticoids	59.3%	57.3%	66.2%	55.1%	58.6%	56.3%	60.4%	52.8%	48.8%
BTK inhibitor	6.8%		1.8%	28.0%	4.4%				0.6%
Venetoclax	3.0%		0.3%	7.5%	0.8%		1.5%		40.7%
“-lidomide”	9.1%		2.4%	0.2%	47.7%	0.3%	4.1%		1.7%
Proteosome inhibitor	6.7%		0.2%		40.3%		0.2%		
MAB for myeloma	3.4%		0.1%	0.1%	20.2%				
Hydroxyurea	5.7%		0.2%	0.2%		22.1%	9.7%	10.6%	12.8%
Methotrexate	3.8%	1.9%	7.1%	3.1%	2.2%	1.7%	3.3%	2.8%	3.5%
JAK2 inhibitor	1.3%			0.1%		3.3%	5.0%	2.2%	5.2%
BCR-ABL inhibitor	2.5%		0.1%	0.1%	0.1%	0.2%	0.6%	77.2%	2.9%
Lymphocyte-depleting	13.4%	6.4%	29.8%	15.4%	7.5%	1.2%	1.9%	1.1%	3.5%
Cytotoxic chemotherapy	18.8%	19.1%	29.8%	12.4%	23.5%	4.5%	15.3%	6.7%	44.8%
IS non-hematologic	4.5%	5.7%	4.6%	1.7%	3.8%	5.8%	5.6%	4.4%	14.0%
**Treatment combinations**									
Combination that includes both GC and non-GC	31.1%	21.7%	34.9%	28.4%	42.8%	19.8%	24.7%	43.3%	33.7%
GC only	28.2%	35.7%	31.3%	26.7%	15.8%	36.5%	35.7%	9.4%	15.1%
Non-GC only	14.8%	5.7%	7.6%	14.8%	23.5%	13.5%	12.0%	41.1%	33.7%
No medication	25.9%	36.9%	26.2%	30.2%	17.9%	30.2%	27.6%	6.1%	17.4%
**Age**									
Mean (SD)	70.9 (11.57)	61.4 (13.02)	71.1 (11.27)	74.3 (8.98)	72.4 (9.39)	65.1 (13.54)	76.5 (10.11)	68.2 (11.20)	69.5 (11.06)
**Sex**									
F	5.0%	12.1%	5.1%	2.0%	3.3%	9.7%	2.9%	4.4%	5.8%
M	95.0%	87.9%	94.9%	98.0%	96.7%	90.3%	97.1%	95.6%	94.2%
**Period of dominant variant**									
Delta	18.0%	17.2%	17.4%	18.7%	18.1%	17.0%	19.5%	18.9%	19.8%
Omicron A	38.7%	46.5%	38.4%	39.2%	39.1%	38.7%	35.5%	36.1%	40.7%
Omicron B	40.5%	33.8%	41.3%	39.1%	40.1%	41.4%	42.7%	42.2%	36.0%
Pre-delta	2.8%	2.5%	2.8%	3.0%	2.7%	2.8%	2.3%	2.8%	3.5%
**Immunity-related**									

© 2024 Anand ST et al. *JAMA Network Open.*

	All	HL	NHL	CLL	PC	MPD	MDS	CML	AML
Boosted	56.6%	43.9%	60.1%	61.4%	61.6%	44.9%	55.4%	56.7%	52.9%
Infection before vaccine	4.3%	3.8%	3.8%	3.5%	4.1%	6.1%	4.4%	2.2%	6.4%
**Comorbidities**									
Alzheimer's / dementia	7.1%	2.5%	5.5%	6.7%	7.0%	8.3%	13.5%	5.6%	6.4%
Chronic kidney disease	30.3%	20.4%	26.3%	27.4%	39.5%	27.7%	39.0%	32.8%	29.1%
COPD / bronchiectasis	18.4%	10.2%	18.8%	16.7%	16.8%	20.5%	23.7%	17.2%	16.9%
Diabetes	38.4%	28.0%	39.6%	37.7%	42.2%	34.5%	41.3%	39.4%	34.9%
Heart failure	12.9%	11.5%	11.9%	10.4%	15.4%	11.3%	23.6%	10.0%	9.9%
Mobility impairments	2.1%	3.2%	1.4%	10.4%	2.0%	3.8%	3.1%	2.8%	0.6%
MS / transverse myelitis	0.6%	1.9%	0.2%	0.6%	0.6%	1.0%	0.6%	0.6%	0.6%
Peripheral vascular disease	8.7%	1.9%	7.7%	8.0%	9.3%	10.1%	13.5%	6.1%	5.2%
Pressure / chronic ulcers	4.2%	1.9%	3.1%	3.3%	4.6%	6.5%	5.8%	2.8%	2.3%
Schizophrenia / psychosis	2.5%	1.9%	2.1%	1.9%	2.7%	3.7%	2.7%	1.1%	1.7%
**Comorbidity score**									
Mean (SD)	1.16 (1.19)	0.74 (1.03)	1.10 (1.14)	1.07 (1.14)	1.30 (1.18)	1.13 (1.19)	1.55 (1.42)	1.11 (1.20)	1.03 (1.10)
**Treatment for SARS-CoV-2**									
Oral antiviral	10.7%	8.9%	12.1%	12.8%	9.9%	8.2%	9.1%	9.4%	11.6%
MAB	10.5%	10.2%	10.0%	13.5%	11.1%	7.3%	9.7%	12.2%	12.8%

HL = Hodgkin's lymphoma. NHL = non-Hodgkin's lymphoma. CLL = chronic lymphocytic leukemia. PC = plasmacytoid malignancy (myeloma or Waldenstrom's). MPD = myeloproliferative disorder without a diagnosis of MDS or CML. MDS = myelodysplastic syndromes. CML = chronic lymphocytic leukemia. AML = acute myeloblastic leukemia. BTK = Bruton tyrosine kinase. "-lidomide" = thalidomide, lenalidomide, pomalidomide. MAB = monoclonal antibodies. IS = immune-suppressive medication. GC = glucocorticoid. COPD = chronic obstructive pulmonary disease. MS = multiple sclerosis.

© 2024 Anand ST et al. *JAMA Network Open.*

eTable 11. Clinical characteristics of patients with severe COVID-19 after vaccination, by subgroup of hematologic malignancy.

N = 6122	All	HL	NHL	CLL	PC	MPD	MDS	CML	AML
N	1301	19	361	275	234	195	146	28	43
**Medications**									
Glucocorticoids	67.4%	42.1%	73.7%	68.0%	62.8%	65.6%	66.4%	75.0%	53.5%
BTK inhibitor	9.2%		1.9%	36.4%	5.6%				
Venetoclax	3.5%		0.6%	7.3%	1.3%		0.7%		44.2%
“-lidomide”	11.6%		3.9%	0.7%	55.6%	0.5%	2.7%		
Proteosome inhibitor	9.8%		0.3%		53.4%		0.7%		
MAB for myeloma	5.1%				28.2%				
Hydroxyurea	5.1%		0.6%	0.7%		16.9%	11.6%	17.9%	16.3%
Methotrexate	4.7%		9.1%	5.5%	0.9%	1.0%	4.8%	3.6%	2.3%
JAK2 inhibitor	1.5%					4.1%	5.5%	7.1%	4.7%
BCR-ABL inhibitor	1.7%				0.4%		0.7%	64.3%	4.7%
Lymphocyte-depleting	17.4%	10.5%	39.3%	18.9%	8.1%	2.1%	2.1%	7.1%	4.7%
Cytotoxic chemotherapy	24.3%	21.1%	39.6%	15.3%	26.9%	8.7%	15.1%	17.9%	46.5%
IS non-hematologic	5.1%	5.3%	5.0%	2.6%	4.7%	7.2%	5.5%	14.3%	7.0%
**Treatment combinations**									
Combination that includes both GC and non-GC	39.9%	21.1%	44.3%	42.6%	50.9%	24.1%	26.7%	64.3%	34.9%
GC only	27.5%	21.1%	29.4%	25.5%	12.0%	41.5%	39.7%	10.7%	18.6%
Non-GC only	14.0%	10.5%	9.1%	12.4%	25.6%	9.7%	11.0%	21.4%	27.9%
No medication	18.6%	47.4%	17.2%	19.6%	11.5%	24.6%	22.6%	3.6%	18.6%
**Age**									
Mean (SD)	75.3 (10.06)	63.3 (12.49)	75.0 (9.77)	77.3 (9.06)	74.5 (9.33)	72.1 (11.76)	79.7 (8.41)	75.2 (11.06)	74.9 (7.44)
**Sex**									
F	3.5%	10.5%	3.9%	2.9%	1.7%	4.6%	2.7%	7.1%	4.7%
M	96.5%	89.5%	96.1%	97.1%	98.3%	95.4%	97.3%	92.9%	95.4%
**Period of dominant variant**									
Delta	25.4%	21.1%	21.6%	22.9%	34.2%	25.1%	27.4%	25.0%	23.3%
OmicronA	34.3%	42.1%	34.9%	38.9%	32.1%	31.3%	32.9%	25.0%	32.6%
OmicronB	36.5%	31.6%	39.0%	34.2%	30.3%	41.5%	36.3%	42.9%	39.5%
Pre-delta	3.8%	5.3%	4.4%	4.0%	3.4%	2.1%	3.4%	7.1%	4.7%
**Immunity-related**									
Boosted	49.9%	26.3%	54.9%	55.6%	48.3%	40.0%	44.5%	53.6%	51.2%
Infection before vaccine	3.2%	5.3%	3.3%	1.8%	3.0%	4.1%	4.1%	3.6%	4.7%
**Comorbidities**									

© 2024 Anand ST et al. *JAMA Network Open.*

N = 6122	All	HL	NHL	CLL	PC	MPD	MDS	CML	AML
Alzheimer's / dementia	13.3%	15.8%	10.8%	12.4%	10.3%	21.0%	17.1%	14.6%	7.0%
Chronic kidney disease	40.4%	36.8%	37.1%	32.7%	53.0%	41.5%	43.2%	42.9%	32.6%
COPD / bronchiectasis	28.4%	10.5%	31.0%	22.9%	24.4%	38.0%	30.1%	28.6%	20.9%
Diabetes	46.9%	36.8%	48.5%	41.8%	55.1%	46.7%	45.2%	39.3%	37.2%
Heart failure	22.8%	21.1%	23.0%	17.1%	23.9%	26.2%	29.5%	25.0%	14.0%
Mobility impairments	3.8%	5.3%	2.8%	1.5%	3.9%	9.2%	3.4%	7.1%	
MS / transverse myelitis	0.4%	5.3%		0.4%	0.4%	1.0%			
Peripheral vascular disease	14.0%		12.7%	14.2%	16.2%	12.8%	18.5%	14.3%	7.0%
Pressure / chronic ulcers	7.7%	5.3%	6.7%	5.1%	8.1%	12.8%	9.6%	10.7%	
Schizophrenia / psychosis	2.9%		1.9%	1.8%	3.9%	6.2%	2.7%		2.3%
**Comorbidity score**									
Mean (SD)	1.66 (1.39)	1.21 (1.18)	1.63 (1.37)	1.41 (1.35)	1.83 (1.33)	1.86 (1.46)	1.84 (1.55)	1.64 (1.34)	1.19 (1.18)
**Treatment for SARS-CoV-2**									
Oral antiviral	1.9%		2.8%	1.5%	2.6%	1.0%	1.4%		2.3%
MAB	6.4%	10.5%	7.8%	8.4%	6.4%	3.6%	3.4%	3.6%	4.7%

HL = Hodgkin's lymphoma. NHL = non-Hodgkin's lymphoma. CLL = chronic lymphocytic leukemia. PC = plasmacytoid malignancy (myeloma or Waldenstrom's). MPD = myeloproliferative disorder without a diagnosis of MDS or CML. MDS = myelodysplastic syndromes. CML = chronic lymphocytic leukemia. AML = acute myeloblastic leukemia. BTK = Bruton tyrosine kinase. "-lidomide" = thalidomide, lenalidomide, pomalidomide. MAB = monoclonal antibodies. IS = immune-suppressive medication. GC = glucocorticoid. COPD = chronic obstructive pulmonary disease. MS = multiple sclerosis.

© 2024 Anand ST et al. *JAMA Network Open.*

eAppendix

Timing of initial vaccination, infection, and diagnosis of malignancy

The initial vaccination series had been completed by April 30, 2021, in 83% of patients. The time from initial vaccination to infection was similar among patients with non-severe or severe COVID-19, either among boosted (median 396 days, interquartile range 309 – 482, for non-severe; median 385 days, interquartile range 309 – 486, for severe) or unboosted patients (median 242 days, interquartile range 155 – 314, for non-severe; median 224 days, interquartile range 153 – 304 for severe). The first use of an ICD-10 code for hematologic malignancy occurred on or before September 30, 2022, in 5852/6122 (95.6%) of patients.

Sub-analyses limited to the omicron B period

Because use of oral antivirals was restricted to the omicron B period, we performed an additional analysis limited to the 2480 patients infected between March 1 and September 30, 2022 (**eTable 12**).

We then further limited this subcohort to the 925 patients who had received a booster vaccine and had good evidence for having active hematologic malignancy, based on treatment with non-glucocorticoid immune-suppressive or antineoplastic drugs. Oral antivirals were used in 227/925 (22.8%) patients, with severe disease developing in 12/227 (5.3%) cases. Among the 643 patients who received no oral antiviral treatment or MAB, 181 (28.1%) either had severe COVID-19 at initial evaluation or developed it subsequently, a distinction that could not be made. Classification of these 643 patients into 16 bins by age ranges and comorbidity scores suggested that rates of severe COVID-19 increased most prominently with age > 80 or comorbidity score > 2 (**eTable 13**). Although these data are exploratory due to the small numbers of cases in each bin and inability to adjust for other variables, the pattern was similar to that seen among the 571 patients infected in the omicron A period (infection December 16, 2021, through February 28, 2022) who had actively treated hematologic malignancy, had been boosted, and did not receive antiviral treatment (**eTable 13**).

© 2024 Anand ST et al. *JAMA Network Open.*

eTable 12. Clinical and demographic data for the subcohort of 2480 patients with hematologic malignancies and SARS-CoV-2 infection after vaccination between March 1 and September 30, 2022 (Omicron B), stratified by severity, and results of multivariable logistic regression analyses with severe versus non-severe infection as the outcome.

N = 2480	Severity		% Severe (19.2%)	Multivariable models, aOR (CI), P	
	Non-severe (N = 2005)	Severe (N = 475)		Individual comorbidities	Comorbidity Score
**Medications**					
Glucocorticoids	1101 (54.9%)	328 (69.1%)	23.0%		
BTK inhibitor	122 (6.1%)	35 (7.4%)	22.3%		
Venetoclax	45 (2.2%)	16 (3.4%)	26.2%		
"-lidomide"	165 (8.2%)	47 (9.9%)	22.2%		
Proteosome inhibitor	116 (5.8%)	45 (9.5%)	28.0%		
MAB for myeloma	56 (2.8%)	23 (4.8%)	29.1%		
Hydroxyurea	121 (6.0%)	29 (6.1%)	19.3%		
Methotrexate	79 (3.9%)	24 (5.1%)	23.2%		
JAK2 inhibitor	24 (1.2%)	9 (1.9%)	27.3%		
BCR-ABL inhibitor	54 (2.7%)	8 (1.7%)	12.9%		
Lymphocyte-depleting	236 (11.8%)	75 (15.8%)	24.1%		
Cytotoxic chemotherapy	327 (16.3%)	116 (24.4%)	26.2%		
IS non-hematologic	88 (4.4%)	22 (4.6%)	20.0%		
**Treatment group**					
Combination that includes both GC and non-GC	557 (27.8%)	180 (37.9%)	24.4%	2.56 (1.87-3.50), <0.001	2.63 (1.93-3.58), <0.001
GC only	544 (27.1%)	148 (31.2%)	21.4%	1.83 (1.33-2.53), 0.44	1.93 (1.41-2.65), 0.26
Non-GC only	305 (15.2%)	69 (14.5%)	18.5%	1.81 (1.25-2.62), 0.63	1.80 (1.25-2.61), 0.76
No medication	599 (29.9%)	78 (16.4%)	11.5%	Referent	Referent
**Disease group**					
Lymphoid	1335 (66.6%)	312 (65.7%)	18.9%		
Myeloid	670 (33.4%)	163 (34.3%)	19.6%		
**Disease Class**					

© 2024 Anand ST et al. *JAMA Network Open.*

N = 2480	Severity		% Severe	Multivariable models, aOR (CI), P	
	Non-severe (N = 2005)	Severe (N = 475)	(19.2%)	Individual comorbidities	Comorbidity Score
AML	45 (2.2%)	17 (3.6%)	27.4%		
CLL	378 (18.9%)	94 (19.8%)	19.9%		
CML	64 (3.2%)	2 (2.5%)	15.8%		
HL	47 (2.3%)	6 (1.3%)	11.3%		
MDS	168 (8.4%)	53 (11.2%)	24.0%		
MPD	393 19.6%)	81 (17.1%)	17.1%		
NHL	574 (28.6%)	141 (29.7%)	19.7%		
PC	336 (16.8%)	71 (15.0%)	17.4%		
**Age**					
Mean (SD), aOR per year	70.90 (11.61)	75.88 (10.50)		1.04 (1.03-1.06), <0.001	1.04 (1.03-1.06), <0.001
**Gender**					
F	102 (5.1%)	26 (5.5%)	20.3%	2.14 (1.30-3.54), 0.003	2.12 (1.28-3.50), 0.003
M	1903 (94.9%)	449 (94.5%)	19.1%	Referent	Referent
**Vaccine Type**					
Janssen	76 (3.8%)	23 (4.8%)	23.3%		
Moderna	993 (49.5%)	220 (46.3%)	18.1%		
Pfizer	936 (46.7%)	232 (48.8%)	19.9%		
**Immunity-related**					
Boosted	1598 (79.7%)	363 (76.4%)	18.5%	0.73 (0.56-0.94), 0.02	0.72 (0.55-0.93), 0.01
No booster	407 (20.3%)	112 (23.6%)	21.6%	Referent	Referent
Infection before vaccine	70 (3.5%)	7 (1.5%)	9.1%		
**Comorbidities**					
Alzheimer’s / dementia	127 (6.3%)	72 (15.2%)	36.2%	1.61 (1.14-2.27), 0.007	
Chronic kidney disease	552 (27.5%)	177 (37.3%)	24.3%	0.96 (0.75-1.24), 0.77	
COPD / bronchiectasis	300 (15.0%)	145 (30.5%)	32.6%	1.90 (1.46-2.46), <0.001	
Diabetes	709 (35.4%)	225 (47.4%)	24.1%	1.53 (1.21-1.92), <0.001	

© 2024 Anand ST et al. *JAMA Network Open.*

N = 2480	Severity		% Severe (19.2%)	Multivariable models, aOR (CI), P	
	Non-severe (N = 2005)	Severe (N = 475)		Individual comorbidities	Comorbidity Score
Heart failure	199 (9.9%)	103 (21.7%)	34.1%	1.54 (1.14-1.10), 0.006	
Liver / cirrhosis	151 (7.5%)	45 (9.5%)	23.0%		
Mobility impairments	36 (1.8%)	4 (5.1%)	40.0%		
MS / transverse myelitis	8 (0.4%)	0 (0.0%)			
Peripheral vascular disease	148 (7.4%)	64 (13.5%)	30.2%	1.17 (0.82-1.66), 0.39	
Pressure / chronic ulcers	65 (3.2%)	41 (8.6%)	38.7%		
Schizophrenia / psychosis	1 (2.0%)	11 (2.3%)	21.2%		
**Comorbidity Score (0-6)**					
Mean (SD), aOR per point	1.01 (1.14)	1.65 (1.36)			1.37 (1.26-1.49), <0.001
**Treatment for SARS-CoV-2**					
Oral antiviral	518 (25.8%)	20 (4.2%)	3.7%	0.12 (0.08-0.20), <0.001	0.12 (0.08-0.20), <0.001
Nirmatrelvir / ritonavir	370 (18.5%)	12 (2.5%)	3.1%		
Molnupiravir	149 (7.4%)	8 (1.7%)	5.1%		
MAB	100 (5.0 %)	17 (3.6%)	14.5%	0.43 (0.25-0.74), 0.002	0.43 (0.25-0.74), 0.002
No antiviral	1395 (69.6%)	439 (92.4%)	23.9%	Referent	Referent

aOR = adjusted odds ratio. CI = 95% confidence interval. HL = Hodgkin's lymphoma. NHL = non-Hodgkin's lymphoma. CLL = chronic lymphocytic leukemia. PC = plasmacytoid malignancy (myeloma or Waldenstrom's). MPD = myeloproliferative disorder without a diagnosis of MDS or CML. MDS = myelodysplastic syndromes. CML = chronic lymphocytic leukemia. AML = acute myeloblastic leukemia. BTK = Bruton tyrosine kinase. "-lidomide" = thalidomide, lenalidomide, pomalidomide. MAB = monoclonal antibodies. IS = immune-suppressive medication. GC = glucocorticoid. COPD = chronic obstructive pulmonary disease. MS = multiple sclerosis

© 2024 Anand ST et al. *JAMA Network Open.*

eTable 13. Numbers and percentages of patients with severe COVID-19, stratified by age and number of specified comorbidities. Numerators = numbers of patients with severe COVID-19; denominators = numbers of patients with infection of any severity. The subcohort was limited to patients who did not receive antivirals, who had received booster vaccination, and who had active hematologic malignancy, based on recent treatment with non-glucocorticoid immune-suppressive or antineoplastic drugs.

	**Number of Comorbidities**			
**Age (years)**	0	1	2	≥ 3
**Omicron B (March 1 – September 30, 2022)**				
< 60	4/35 (11.4%)	4/14 (28.6%)	2/10 (20.0%)	0/1 (0.0%)
60-69	8/37 (21.6%)	10/36 27.8%)	6/25 (24.0%)	7/11 (63.6%)
70-79	18/111 (16.2%)	28/106 (26.4%)	16/77 (20.8%)	22/49 (44.9%)
≥ 80	8/33 (24.2%)	14/41 (34.1%)	17/35 (48.6%)	8/22 (36.4%)
**Omicron A (December 16, 2021 – February 28, 2022)**				
< 60	4/42 (9.5%)	3/13 (23.1%)	2/10 (20.0%)	1/5 (20.0%)
60-69	11/66 (16.7%)	5/30 16.7%)	4/27 (14.8%)	8/19 (42.1%)
70-79	17/86 (19.8%)	18/87 (20.7%)	27/69 (39.1%)	12/41 (29.3%)
≥ 80	9/25 (36.0%)	8/20 (40.0%)	4/14 (28.6%)	14/17 (82.4%)
**Combined (December 16, 2021 – September 30, 2022)**				
< 60	8/77 (10.4%)	7/27 (25.9%)	4/20 (20.0%)	1/6 (16.7%)
60-69	19/103 (18.4%)	15/66 (22.7%)	10/52 (19.2%)	15/30 (50.0%)
70-79	35/197 (17.8%)	46/193 (23.8%)	43/146 (29.5%)	34/90 (37.8%)
≥ 80	17/58 (29.3%)	22/61 (36.1%)	21/49 (42.9%)	22/39 (56.4%)

Comorbidities: Alzheimer's/dementia, chronic kidney disease, chronic obstructive pulmonary disease or bronchiectasis, diabetes, heart failure, peripheral vascular disease

© 2024 Anand ST et al. *JAMA Network Open.*

eTable 14. Clinical and demographic data for the full cohort of 6122 patients with hematologic malignancies and SARS-CoV-2 infection after vaccination, and the 214 patients who died 2-28 days after diagnosis of infection. Numbers were too small to attempt multivariable analysis. Deaths were not adjudicated by chart review to determine whether they were COVID-19-related.

	Total in cohort (N = 6122)	Deaths (N = 214)	% Died (3.5%)
**Disease Class**			
AML	172 (2.8%)	8 (3.7%)	4.7%
CLL	1206 (19.7%)	59 (27.6%)	4.9%
CML	180 (2.9%)	7 (3.3%)	3.9%
HL	157 (2.6%)	3 (1.4%)	1.9%
MDS	518 (8.5%)	19 (8.9%)	3.7%
MPD	1144 (18.7%)	26 (12.2%)	2.3%
NHL	1731 (28.3%)	55 (25.7%)	3.2%
PC	1014 (61.6%)	37 (17.3%)	3.6%
**Disease group**			
Lymphoid	4108 (67.1%)	154 (72.0%)	3.7%
Myeloid	2014 (32.9%)	60 (28.0%)	3.0%
**Treatment group**			
Combination that includes both GC and non-GC	1905 (31.1%)	93 (43.5%)	4.9%
Glucocorticoids only	1725 (28.2%)	38 (17.8%)	2.2%
Non-glucocorticoids only	1588 (25.1%)	30 (14.0%)	1.9%
No medication	904 (14.8%)	53 (24.8%)	5.9%
**Age**			
Mean (SD)	70.9 (11.57)	78.3 (8.66)	
**Sex**			
F	308 (5.0%)	5 (2.3%)	1.6%
M	5814 (95.0%)	209 (97.7%)	3.6%
**Period of dominant variant**			
Delta	1102 (18.0%)	65 (30.4%)	5.9%
Omicron A	2369 (38.7%)	79 (36.9%)	3.3%
Omicron B	2480 (40.5%)	68 (31.8%)	2.7%
Pre-delta	171 (2.8%)	2 (0.9%)	1.2%
**Immunity-related**			

© 2024 Anand ST et al. *JAMA Network Open.*

	Total in cohort (N = 6122)	Deaths (N = 214)	% Died (3.5%)
Boosted	3468 (56.6%)	103 (48.1%)	3.0%
No boost	2654 (43.4%)	111 (51.9%)	4.2%
Infection before vaccinated	263 (4.3%)	2 (0.9%)	0.8%
**Comorbidities**			
Alzheimer's / dementia	437 (7.1%)	30 (14.0%)	6.9%
Chronic kidney disease	1857 (13.3%)	89 (41.6%)	4.8%
COPD / bronchiectasis	1128 (18.4%)	54 (25.2%)	4.8%
Diabetes	2353 (38.4%)	104 (48.6%)	4.4%
Heart failure	792 (12.9%)	61 (28.5%)	7.7%
Liver / cirrhosis	540 (8.8%)	29 (13.6%)	5.4%
Mobility impairments	127 (2.1%)	3 (1.4%)	2.4%
MS / transverse myelitis	35 (0.6%)	0 (0.0%)	0.0%
Peripheral vascular disease	531 (8.7%)	38 (17.8%)	7.2%
Pressure / chronic ulcers	257 (4.2%)	18 (8.4%)	7.0%
Schizophrenia / psychosis	150 (2.5%)	6 (2.8%)	4.0%
**Comorbidity Score**			
Mean (SD)	1.16 (1.19)	1.76 (1.45)	
**Treatment for SARS-CoV-2**			
Oral antiviral	655 (10.7%)	3 (1.4%)	0.5%
Nirmatrelvir / ritonavir	467 (7.6%)	2 (0.9%)	0.4%
Molnupiravir	189 (3.1%)	1 (0.5%)	0.5%
MAB	641 (10.5%)	6 (2.8%)	0.9%
No antiviral	4864 (79.5%)	205 (95.8%)	4.2%

HL = Hodgkin's lymphoma. NHL = non-Hodgkin's lymphoma. CLL = chronic lymphocytic leukemia. PC = plasmacytoid malignancy (myeloma or Waldenstrom's). MPD = myeloproliferative disorder without a diagnosis of MDS or CML. MDS = myelodysplastic syndromes. CML = chronic lymphocytic leukemia. AML = acute myeloblastic leukemia. MAB = monoclonal antibodies. IS = immune-suppressive medication. GC = glucocorticoid. COPD = chronic obstructive pulmonary disease. MS = multiple sclerosis.

© 2024 Anand ST et al. *JAMA Network Open.*
