# Supplementary material for: Severe COVID-19 in Vaccinated Adults With Hematologic Cancers in the Veterans Health Administration
Source: JAMA Netw Open. 2024 Feb 23;7(2):e240288. doi: 10.1001/jamanetworkopen.2024.0288 (PMC10891464; doi:10.1001/jamanetworkopen.2024.0288)
Supplement: Supplement 2. — Data Sharing Statement [file jamanetwopen-e240288-s002.pdf]

Data Sharing Statement

Anand. Severe COVID-19 in Vaccinated Adults With Hematologic Cancers in the Veterans Health Administration. *JAMA Netw Open*. Published February 23, 2024. doi:10.1001/jamanetworkopen.2024.0288

Data
Data available: No

Additional Information
Explanation for why data not available: Per VA policy, patient-level data cannot be shared except under very limited circumstances. This statement is included at the end of the manuscript, after the acknowledgments. A data dictionary is provided giving the definitions for the variables used in the study.
